# The oldest described eurypterid: a giant Middle Ordovician (Darriwilian) megalograptid from the Winneshiek Lagerstätte of Iowa

**DOI:** 10.1186/s12862-015-0443-9

**Published:** 2015-09-01

**Authors:** James C. Lamsdell, Derek E. G. Briggs, Huaibao P. Liu, Brian J. Witzke, Robert M. McKay

**Affiliations:** Department of Geology and Geophysics, Yale University, 210 Whitney Avenue, New Haven, CT 06511 USA; Yale Peabody Museum of Natural History, Yale University, New Haven, CT 06511 USA; Iowa Geological Survey, IIHR-Hydroscience & Engineering, University of Iowa, 340 Trowbridge Hall, Iowa City, IA 52242 USA; Department of Earth and Environmental Sciences, University of Iowa, 121 Trowbridge Hall, Iowa City, IA 52242 USA

## Abstract

**Background:**

Eurypterids are a diverse group of chelicerates known from ~250 species with a sparse Ordovician record currently comprising 11 species; the oldest fully documented example is from the Sandbian of Avalonia. The Middle Ordovician (Darriwilian) fauna of the Winneshiek Lagerstätte includes a new eurypterid species represented by more than 150 specimens, including some juveniles, preserved as carbonaceous cuticular remains. This taxon represents the oldest described eurypterid, extending the documented range of the group back some 9 million years.

**Results:**

The new eurypterid species is described as *Pentecopterus decorahensis* gen. et sp. nov.. Phylogenetic analysis places *Pentecopterus* at the base of the Megalograptidae, united with the two genera previously assigned to this family by the shared possession of two or more pairs of spines per podomere on prosomal appendage IV, a reduction of all spines except the pair on the penultimate podomere of appendage V, and an ornamentation of guttalate scales, including angular scales along the posterior margin of the dorsal tergites and in longitudinal rows along the tergites. The morphology of *Pentecopterus* reveals that the Megalograptidae are representatives of the derived carcinosomatoid clade and not basal eurypterids as previously interpreted.

**Conclusions:**

The relatively derived position of megalograptids within the eurypterids indicates that most eurypterid clades were present by the Middle Ordovician. Eurypterids either underwent an explosive radiation soon after their origination, or earlier representatives, perhaps Cambrian in age, remain to be discovered. The available instars of *Pentecopterus decorahensis* suggest that eurypterids underwent extreme appendage differentiation during development, a potentially unique condition among chelicerates. The high degree of appendage specialization in eurypterids is only matched by arachnids within chelicerates, supporting a sister taxon relationship between them.

**Electronic supplementary material:**

The online version of this article (doi:10.1186/s12862-015-0443-9) contains supplementary material, which is available to authorized users.

## Background

Eurypterids are a monophyletic group of Paleozoic aquatic arthropods which represent the first major radiation of chelicerates: some 250 species are known from marine to freshwater environments [1]. Eurypterids are relatively common components of Silurian and Devonian Lagerstätten where conditions favor the preservation of their unmineralized cuticle [2]. They are distinctive Paleozoic arthropods, with a fossil record previously known to extend from the Sandbian (Late Ordovician) to the Wuchiapingian (Permian) [1]. The Ordovician record of eurypterids is sparse, however, and the majority of occurrences reported in the literature have been shown to be either misidentifications of other taxa or pseudofossils [3]. Currently, 11 species of Ordovician eurypterid are known, falling into two ecological categories: larger active predators from Laurentia [4–6] and more basal demersal forms from Gondwana and Avalonia [7–9].

Here, we describe a new species of megalograptid eurypterid, *Pentecopterus decorahensis* gen. et sp. nov., from the Middle Ordovician (Darriwilian) Winneshiek Lagerstätte of Decorah, Iowa [10, 11], extending the stratigraphic range of Eurypterida back some 9 million years. The material is exceptionally preserved as organic cuticle remains providing remarkably complete information on the overall morphology as well as details of the microstructure. In addition to abundant adults a limited number of juvenile specimens are present, revealing ontogenetic changes within the species.

This new taxon is important in expanding our limited knowledge of eurypterid cuticular structures. While Holm’s spectacular material of *Eurypterus* cuticle from the Silurian of the Baltic (Saareema) has received attention [12–15], most previous papers have simply reported the occurrence of preserved cuticle [16–19], although there have been a few studies of cuticular structure [20, 21] and chemistry [2, 22]. Ontogenetic data are available for basal eurypterids [23–26]; *Pentecopterus* is the first derived taxon to provide evidence of development. Here we describe the new species and place it within a phylogenetic framework, discussing its significance for the early evolution and postembryonic development of eurypterids.

## Methods

### Material

The majority of the specimens described here were collected from the upper 4 m section of the Winneshiek Shale which was excavated from its only outcrop near Decorah, northeastern Iowa, in 2010. Other samples were collected from blocks eroded during flooding, which are assumed to have been sourced from the uppermost 2–3 m. The material yielded over 5,000 fossil specimens (*n* = 5,354) of which about 6.6 % are eurypterid remains. This number excludes cuticular fragments too small to provide information on the morphology of the eurypterid. Arthropods, which also include phyllocarids (7.9 %) and other bivalved taxa (1.6 %) [27], make up the dominant and most diverse invertebrate group of the Winneshiek fauna. All material described here is accessioned in the Paleontology Repository, Department of Earth and Environmental Sciences, University of Iowa.

Specimens were prepared by using water to disaggregate the matrix and steel periodontal probes and bin angled chisels to remove matrix from the cuticle. Specimens less than 20 mm in dimension were photographed using a Leica DFC420 digital camera attached to a Leica MZ16 stereomicroscope: cuticle free of the matrix was illuminated with light transmitted through the microscope stage. Specimens larger than 20 mm were photographed using a Canon EOS 60D digital camera with a Canon EF-S 60 mm f/2.8 Macro USM lens; cuticle free of the matrix was illuminated with transmitted light generated from a Huion L42 LED light pad. All specimens were imaged dry and with normal light. Image cropping and leveling was carried out using Adobe Photoshop CS5, and interpretive drawings were prepared with Adobe Illustrator CS5, on a MacBook Pro running OS X.

### Geological setting and preservation

In 2005, geologists of Iowa Geological Survey discovered an unusual fossil fauna from the Winneshiek Shale in northeastern Iowa. This fauna is characterized by abundant well-preserved fossils including conodonts, arthropods, possible jawless fish, algae, and plant materials, and represents a new fossil Lagerstätte [10]. Based mainly on the conodont taxa present, the Winneshiek fauna is dated as Middle Ordovician (Darriwilian: 467.3 – 458.4 Ma) in age [10, 11].

The Winneshiek Shale is an 18–27 m thick greenish brown to dark grey laminated sandy shale [28, 29]. It overlies an unnamed stratigraphic unit of thick massive breccia and is in turn disconformably overlain by the St. Peter Sandstone [10, 11]. The Winneshiek Shale crops out only in one locality which is mostly submerged by the Upper Iowa River near Decorah. Bore hole data indicate that the total thickness of the Winneshiek Shale is about 18 m at the outcrop locality, but only the upper 4 m was systematically collected during the excavation.

The Winneshiek Shale is confined to a circular basin about 5.6 km in diameter in the Decorah area. Multiple lines of geological evidence indicate that the circular basin originated from a meteorite impact [11, 29]. The shape and dimension of the impact structure have recently been established by aerial geophysical surveys conducted by the U.S. Geological Survey, and the crater has been named the Decorah Impact Structure. Paleogeographic and paleoenvironmental studies indicate that the crater was located in marginal to nearshore marine conditions, with low-oxygen and possibly brackish water, within tropical southern Laurentia [11, 30, 31]. Rhythmic sandy laminations may indicate a local tidal influence [11, 30, 31]. The Winneshiek fauna is dramatically different from a normal marine shelly fossil fauna, indicating that the restricted environment was inhospitable to typical marine taxa [10, 11].

The eurypterid material comprises partially disarticulated individuals preserved as organic cuticle within fine shale laminations. The cuticle is red- to yellow-brown in color and does not fluoresce under UV light, even though the cuticle of both scorpions [32, 33] and xiphosurids (J. Lamsdell pers. obs.), which bracket eurypterids phylogenetically [34, 35], is known to do so. This lack of fluorescence may be original or due to diagenetic change: previous studies of eurypterid cuticle have found it to be almost identical in structure to that of xiphosurids [36], although we know of no other attempts to determine whether eurypterid cuticle fluoresces. The specimens exhibit patterns typical of eurypterid exuviae in late stages of disarticulation [37], including isolated ventral plates, tergites, and prosomal appendages, and form two-dimensional compressions of the dorsal and ventral surfaces with a fine sediment infill; internal soft tissue is not preserved. Eurypterids have been hypothesized to molt *en masse* [38–40], and accumulations of molts have been reported from a number of sheltered, marginal marine environments [26, 41–43], suggesting that the specimens are exuviae that accumulated within the Decorah crater during molting.

### Institutional abbreviation

SUI, University of Iowa Paleontology Repository, Iowa City, Iowa, USA.

### Terminology

Eurypterid terminology largely follows Tollerton [44] for morphology of the carapace, lateral eyes, prosomal appendages, metastoma, genital appendage, opisthosomal differentiation, telson, and marginal ornamentation; however, the terminology for the ventral plate follows Tetlie et al. [37]. Terminology for prosomal structures and cuticular sculpture, and the labeling of the appendages, follows Selden [14]. Minor modifications to the terminology used in these papers follows Lamsdell [45].

### Phylogenetic analysis

The phylogenetic analysis presented herein is based on an expanded version of the matrix of Lamsdell and Selden [26]. Sampling of the Carcinosomatoidea is increased, with ten species included for the carcinosomatid, megalograptid, and mixopterid clades in addition to the three sampled previously. The genus *Alkenopterus* Størmer, 1974 is also included for the first time, as it has been shown to be a basal representative of the Eurypterina [46] rather than a stylonurine as previously interpreted [47]. Only one carcinosomatoid genus was omitted: *Eocarcinosoma* Caster and Kjellesvig-Waering, 1964, which is known from a single small carapace. The new matrix consists of 158 characters coded for 74 taxa. Additional file 1 includes the matrix and the character descriptions; it has also been deposited in the online MorphoBank database [48] under the project code p2116 and can be accessed from http://morphobank.org/permalink/?P2116.

The analysis was performed using TNT [49] (made available with the sponsorship of the Willi Hennig Society) employing random addition sequences followed by tree bisection-reconnection (TBR) branch swapping (the *mult* command in TNT) with 100,000 repetitions with all characters unordered and of equal weight. Jackknife [50], Bootstrap [51] and Bremer [52] support values were calculated in TNT; the ensemble Consistency, Retention and Rescaled Consistency Indices were calculated in Mesquite 3.02 [53]. Bootstrapping was performed with 50 % character resampling for 5,000 repetitions, and jackknifing by using simple addition sequence and tree bisection-reconnection branch swapping for 5,000 repetitions with 33 % character deletion.

### Nomenclatural acts

This article conforms to the requirements of the amended International Code of Zoological Nomenclature, and hence the new names contained herein are available under that Code. This published work and the nomenclatural acts it contains have been registered in ZooBank, the online registration system for the ICZN. The ZooBank LSIDs (Life Science Identifiers) can be resolved and the associated information viewed through any standard web browser by appending the LSID to the prefix “http://zoobank.org/”. The LSID for this publication is: urn:lsid:zoobank.org:pub:6E58DCAD-B5A8-4552-98B7-FA5585A20499. The journal is identified by ISSN 1471–2148, and has been archived and is available from the following digital repositories: PubMed Central, LOCKSS, INIST, and Koninklijke Bibliotheek.

## Results

### Systematic paleontology

CHELICERATA Heymons, 1901

EURYPTERIDA Burmeister, 1843

EURYPTERINA Burmeister, 1843

DIPLOPERCULATA Lamsdell, Hoşgör and Selden, 2013

CARCINOSOMATOIDEA Størmer, 1934

MEGALOGRAPTIDAE Caster and Kjellesvig-Waering *in* Størmer, 1955

*Pentecopterus* gen. nov.

LSID: *urn:lsid:zoobank.org:act:803A3DD9-AF58-4ADC-AA64-CEA1AA051124*

*Pentecopterus decorahensis* sp. nov.

LSID: *urn:lsid:zoobank.org:act:37E2232A-70D7-48DD-9CE6-90B5214B5A0E*

Figures 1, 2, 3, 4, 5, 6, 7, 8, 9, 10, 11, 12, 13, 14, 15, 16, 17, 18, 19, 23 and 24

**Fig. 1:**
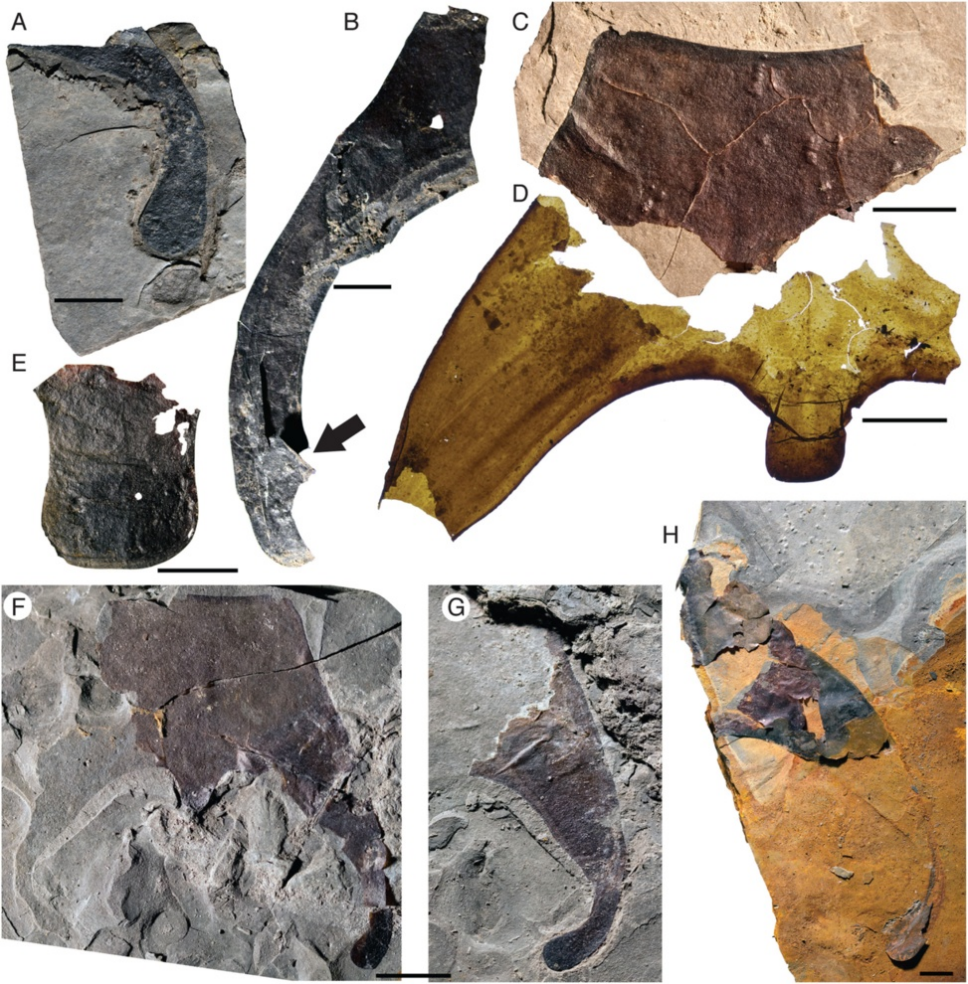
*Pentecopterus decorahensis*, prosomal ventral plate. **a** SUI 139914, posterior lobe of lateral portion of ventral plate. **b** SUI 139978, lateral portion of ventral plate showing carapace locking mechanism (arrowed). **c** SUI 139936, anterior portion of ventral plate including rostrum, retained on shale. **d** SUI 139936, posterior portion of ventral plate shown in Fig. 1c including linguoid projection, removed from sediment. **e** SUI 1139921, linguoid projection. **f** SUI 139917 part, ventral plate. **g** SUI 139917 counterpart, lateral portion of ventral plate. **h** SUI 139916, lateral portion of large ventral plate. Scale bars = 10 mm

**Fig. 2:**
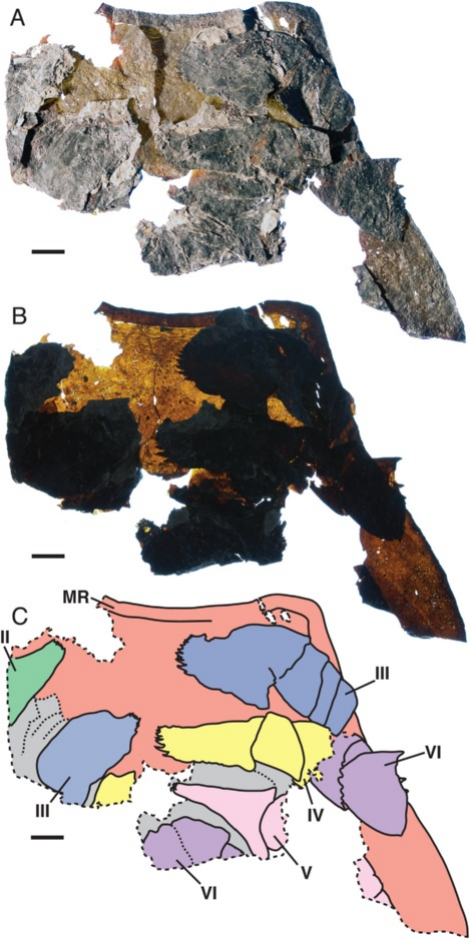
*Pentecopterus decorahensis*, SUI 139979 - prosomal ventral plate, rostrum and proximal limb podomeres. **a** Rostrum and lateral portion of ventral plate with overlying proximal portions of prosomal appendages II–VI, direct light. **b** Transmitted light. **c** Interpretive drawing: red = ventral plate, green = appendage II, blue = appendage III, yellow = appendage IV, pink = appendage V, purple = appendage VI, and gray = ventral carapace cuticle, MR = marginal rim, II–VI = appendages II–VI. Scale bars = 10 mm

**Fig. 3:**
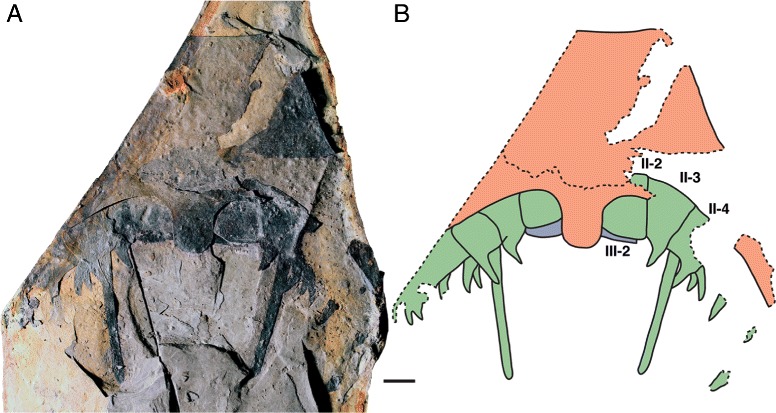
*Pentecopterus decorahensis*, SUI 139941 (holotype) - rostrum and linguoid posterior projection underlain by left and right prosomal appendages II and III. **a** Specimen. **b** Interpretive drawing: red = ventral plate, green = appendage II, and blue = appendage III, II-2–II-4 = appendage II podomeres 2–4, III-2 = appendage III podomere 2. The coxa are angled anteriorly and covered by the ventral plate, which can be peeled back to reveal their position. Scale bar = 10 mm

**Fig. 4:**
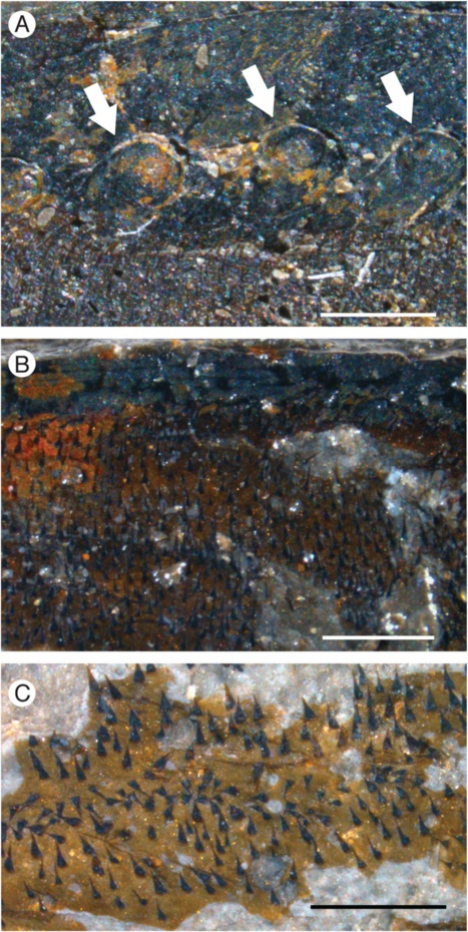
*Pentecopterus decorahensis*, cuticular structures on prosomal ventral plate. **a** SUI 140009, lateral portion of prosomal ventral plate showing row of scales (arrowed) along the inner margin. **b** SUI 140009, prosomal ventral plate with terrace lines and ventral prosomal integument bearing setae. **c** SUI 140011, setae covering ventral prosomal integument. Each figure is oriented with the specimen anterior to the left. Scale bars = 1 mm

**Fig. 5:**
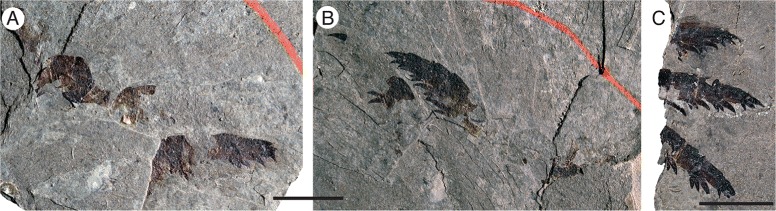
*Pentecopterus decorahensis*, juveniles. **a**, **b** SUI 139963, prosomal appendages III and IV, (**a**) counterpart, (**b**) part. **c** SUI 139965, prosomal appendages II–IV. Scale bars = 10 mm

**Fig. 6:**
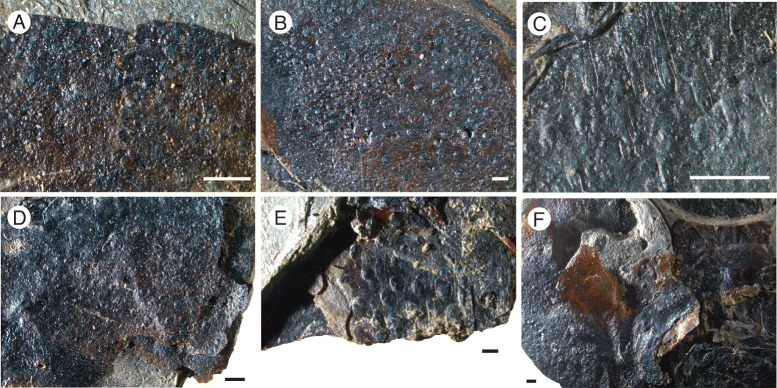
*Pentecopterus decorahensis*, cuticular structures on prosomal appendages. **a** SUI 139963, juvenile appendage showing denticulations of podomere margin and dorsal row of scales. **b** SUI 139951, coxa of appendage III with dense covering of conical scales. **c** SUI 139952, fourth podomere of appendage III showing raised scales with apical follicle. **d** SUI 139913, coxa of appendage V exhibiting dense covering of small scales. **e** SUI 139945, coxa of appendage VI with large scales. **f** SUI 139949, third to fifth podomeres of appendage VI with scattered small scales. Scale bars = 1 mm

**Fig. 7:**
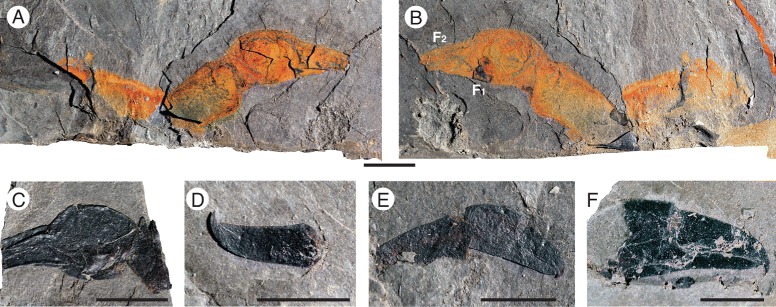
*Pentecopterus decorahensis*, chelicera. **a**, **b** SUI 139952, complete chelicera. **a** Part. **b** Counterpart. **c** SUI 139939, dorsal view of chelicera. **d** SUI 139934, free finger. **e** SUI 139935, complete chelicera. **f** SUI 139983, free and fixed fingers. F_1_ = fixed finger, F_2_ = free finger. Scale bars = 10 mm

**Fig. 8:**
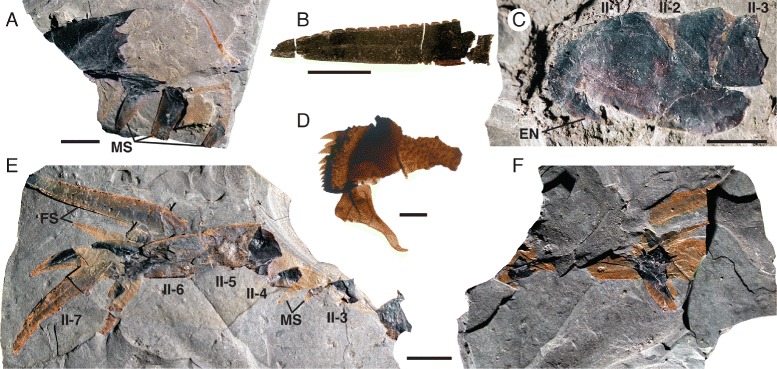
*Pentecopterus decorahensis*, prosomal appendage II. **a** SUI 139919, fragmentary appendage showing moveable spines. **b** SUI 140018, enlarged fixed spine with serration. **c** SUI 139975, coxa with moveable endite and podomeres 2–3. **d** SUI 139969, small coxa with moveable endite. **e**, **f** SUI 139920, podomeres 3–7 showing moveable and fixed spines. All spines are *in situ* except for the large fixed spine originating from the fourth podomere, which is detached and angled anteriorly to the appendage. **e** Part, (**f**) Counterpart. II-1–II-7 = coxa and appendage podomeres 1–7, EN = endite, FS = fixed spines, MS = moveable spines. Scale bars for **a**–**c**, **e**–**f** = 10 mm, for **d** = 1 mm

**Fig. 9:**
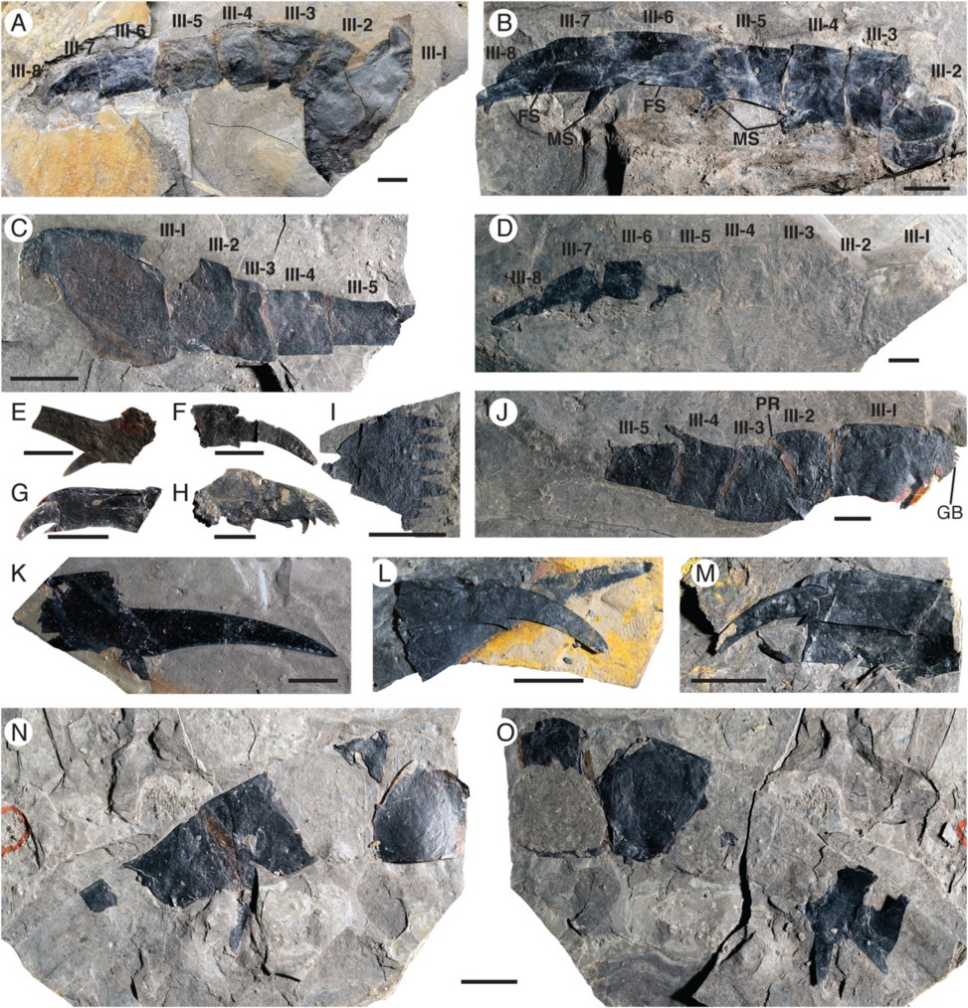
*Pentecopterus decorahensis*, prosomal appendage III. **a** SUI 102857, complete appendage lacking armature. **b** SUI 139953, podomeres 2–8 showing enlargement of fixed lateral spines on more distal podomeres. **c** SUI 139951, podomeres 1–5. **d** SUI 139948, complete appendage, podomeres 1–5 preserved as imprints. **e** SUI 139973, sixth podomere showing moveable and fixed spines. **f** SUI 139990, podomeres 7–8 in ventral view. **g** SUI 140013, podomeres 6–8 in ventral view. **h** SUI 140007, podomeres 6–8 in lateral view. **i** SUI 139925, gnathobase of coxa. **j** SUI 139952, podomeres 1–5. **k** SUI 139922, large podomere 6 displaying fixed spine. **l** SUI 139944, podomeres 7–8 with enlarged fixed spine of podomere 6. **m** SUI 139929, podomeres 7–8 with proximal region of fixed spine of podomere 6. **n**, **o** SUI 139930, podomeres 2–6. **n** Part, **o** Counterpart. III-1–III-8 = podomeres 1–8, *GB* = gnathobase, *FS* = fixed spine, *MS* = moveable spine, *PR* = podomere rotation. Scale bars = 10 mm

**Fig. 10:**
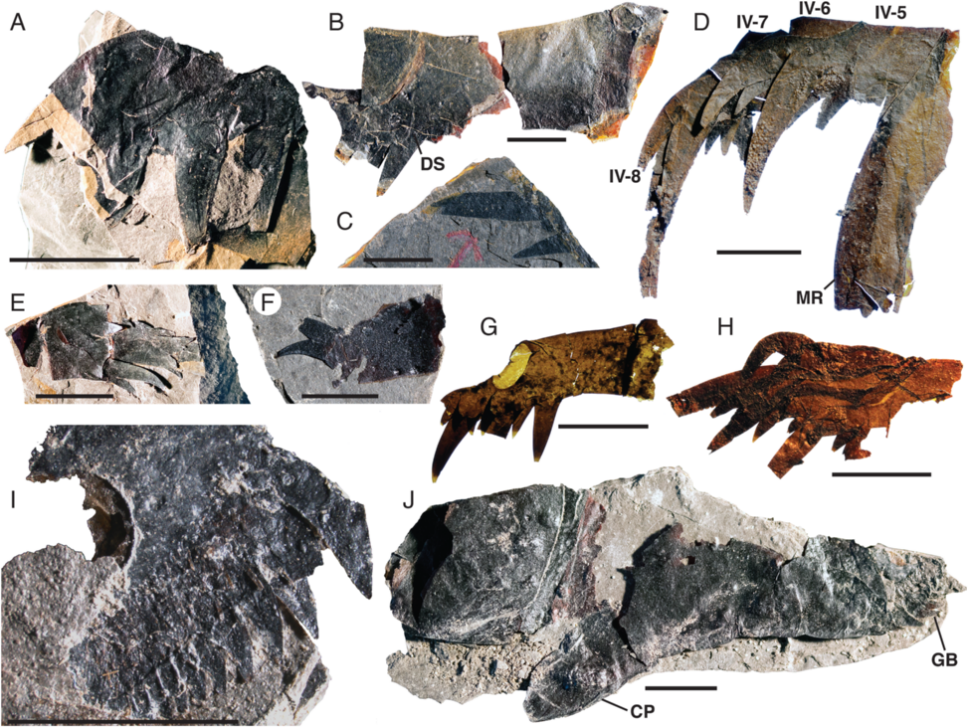
*Pentecopterus decorahensis*, prosomal appendage IV. **a** SUI 139937, fragmentary appendage showing moveable spines. **b** SUI 140000, podomeres 2–3 showing development of spines on the ventral distal podomere margin. **c** SUI 139927, termination of appendage. **d** SUI 139926, most complete example of appendage known, comprising podomeres 5–8, and lateral portion of carapace with marginal rim. **e** SUI 140012, podomeres 6–8. **f** SUI 139928, fourth podomere showing moveable spines. **g** SUI 139938, podomere displaying ventral distal extension and development of marginal spines. **h** SUI 139940, podomeres 6–8. **i** SUI 139959, coxa showing development of ancillary spines surrounding gnathobase. **j** SUI 139946, coxa and second podomere. IV-5–IV-8 = podomeres 5–8, *CP* = coxal projection, *DS* = distal swelling, *GB* = gnathobases, *MR* = marginal rim. Scale bars = 10 mm

**Fig. 11:**
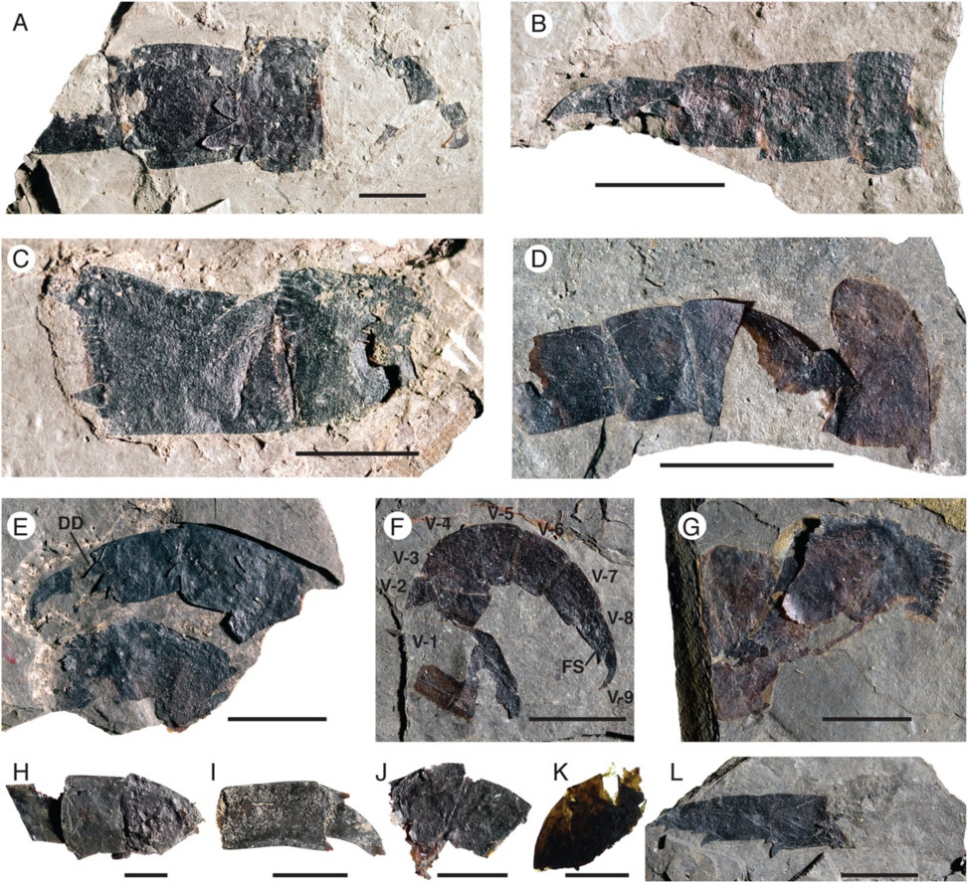
*Pentecopterus decorahensis*, prosomal appendage V. **a** SUI 140014, podomeres 4–7. **b** SUI 139986, podomeres 6–9. **c** SUI 139989, podomeres 3–4. **d** SUI 139942, podomeres 1–5. **e** SUI 139913, coxa and podomeres 6–9 showing distal denticulation of podomeres. **f** SUI 139970, complete appendage. **g** SUI 139924, coxa. **h** SUI 139985, podomeres 4–6. **i** SUI 139912, appendage termination. **j** SUI 139991, podomeres 4–5. **k** SUI 139996, podomeres 4–5. **l** SUI 139968, podomeres 6–9. V-1–V-9 = podomeres 1–9, *DD* = distal denticulations, *FS* = fixed spine. Scale bars = 10 mm

**Fig. 12:**
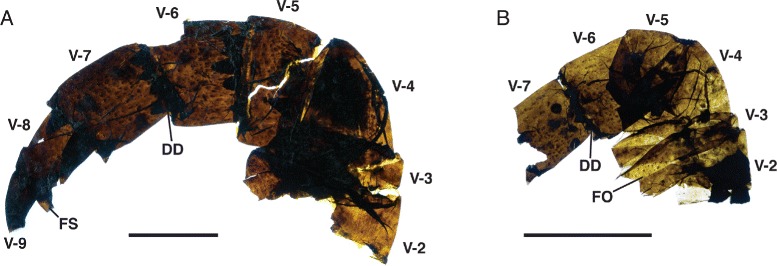
*Pentecopterus decorahensis*, prosomal appendage V. **a** SUI 139998, podomeres 2–9 showing distal podomere serrations and distribution of setal follicles. **b** SUI 140016, podomeres 2–7. V-2–V-9 = podomeres 2–9, *DD* = distal denticulation, *FO* = follicles, *FS* = fixed spine. Scale bars = 10 mm

**Fig. 13:**
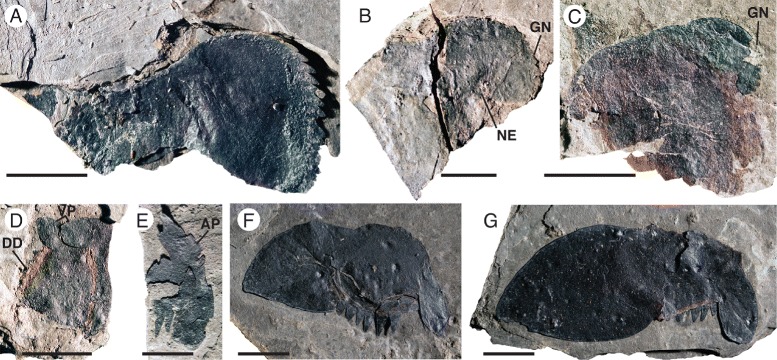
*Pentecopterus decorahensis*, prosomal appendage VI. **a** SUI 139945, coxa. **b** SUI 139987, damaged coxa. **c** SUI 139994, coxa with gnathobase compressed into ‘neck’. **d** SUI 139997, podomeres 5–6 showing distal denticulations. **e** SUI 139993, lateral portion of sixth podomere showing attachment point for preceding podomere. **f** SUI 139961, isolated sixth podomere. **g** SUI 139967, isolated sixth podomere. *AP* = attachment point, *DD* = distal denticulation, *GN* = gnathobase, *NE* = ‘neck’, *VP* = ventral projection. Scale bars = 10 mm

**Fig. 14:**
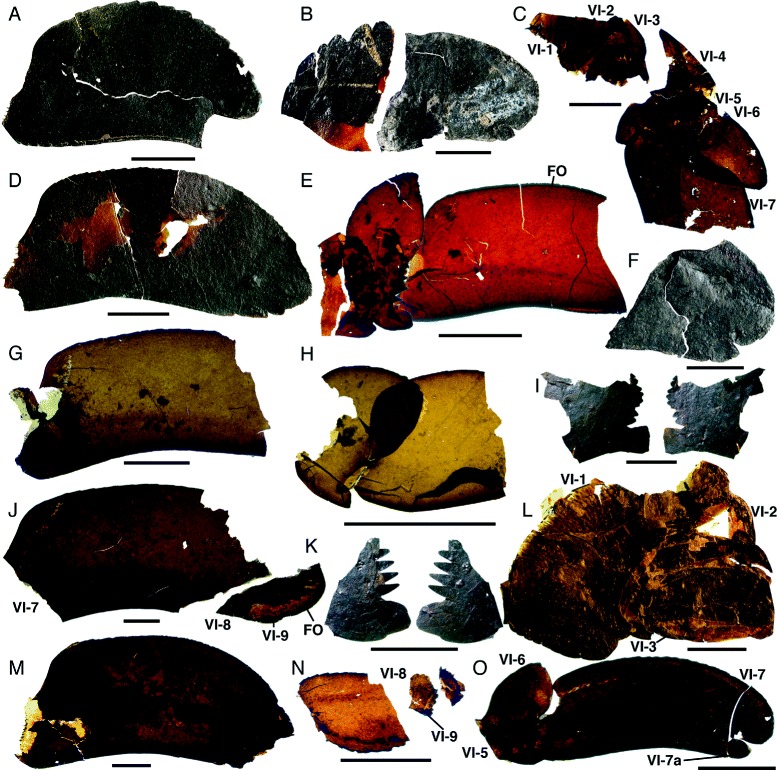
*Pentecopterus decorahensis*, prosomal appendage VI. **a** SUI 139918, seventh podomere showing serrations and anterodistal projection. **b** SUI 139999, podomeres 7–9 showing serrations on proximal region of seventh podomere. **c** SUI 139984, almost complete articulated paddle comprising podomeres 1–7. **d** SUI 139933, podomeres 7–9. **e** SUI 139960, paddle showing articulations between podomeres 5–7 and the distribution of setal follicles. **f** SUI 139992, podomeres 8–9. **g** SUI 139988, seventh podomere. **h** SUI 139995, podomeres 6–7 showing articulation and overlap. **i** SUI 139958, distal portion of sixth podomere shown in both anterior and posterior aspect displaying the continuation of serrations behind the lateral projection. **j** SUI 139981, incomplete seventh podomere and distal portion of eighth showing the insertion of podomere 9. **k** SUI 139966, distal portion of sixth podomere in both anterior and posterior aspect. **l** SUI 139949, podomeres 1–3. **m** SUI 139977, seventh podomere. **n** SUI 139964, podomeres 8–9. **o** SUI 140001, small paddle showing podomeres 6–7 and the positioning of podomere 7a posterior to the anterodistal projection. VI-1–VI-9 = podomeres 1–9, *FO* = follicles. Scale bars = 10 mm

**Fig. 15:**
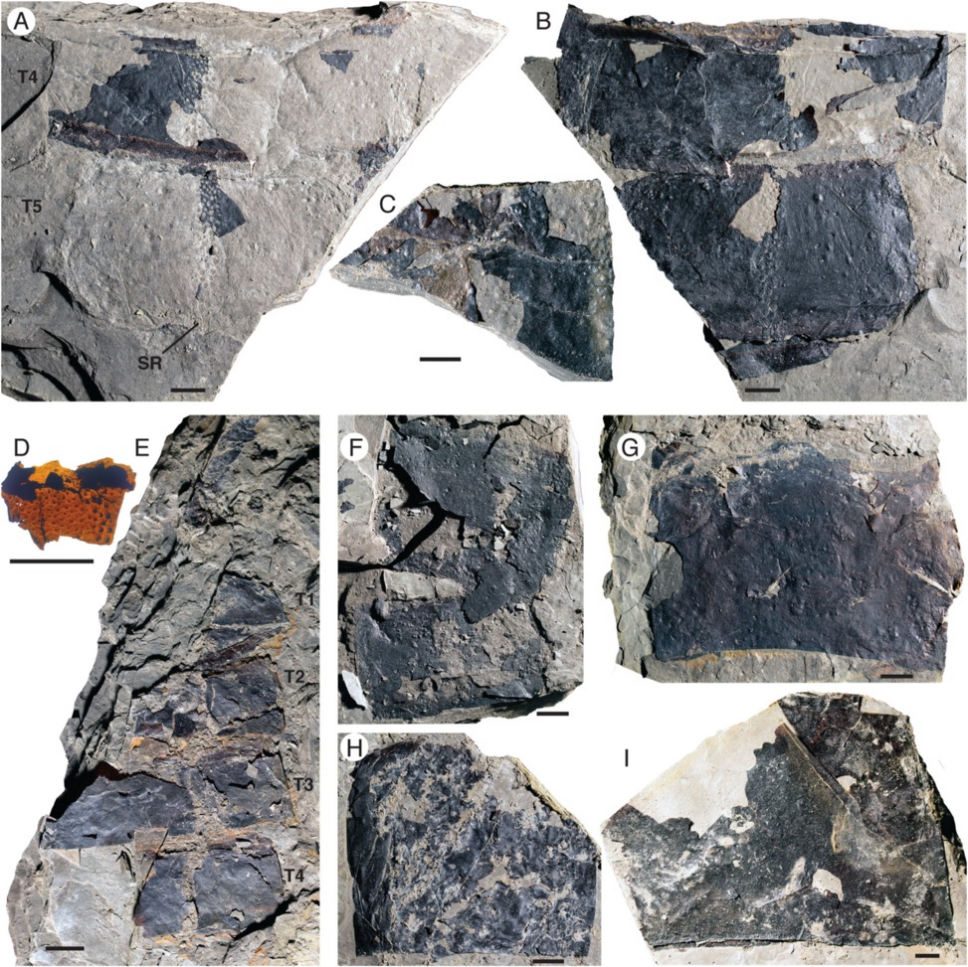
*Pentecopterus decorahensis*, mesosomal tergites. **a**, **b** SUI 139931. **a** Counterpart showing imprint of central scale rows in the sediment. **b** Part, articulated tergites probably representing segments 4–5. **c** SUI 139943, tergites 2–4. **d**, **e** SUI 139947. **d** Counterpart, fragment of cuticle removed from second tergite. **e** Part, tergite 1–4. **f** SUI 140018 part, two tergites in series. **g** SUI 139950, tergite. **h** SUI 139932, sixth tergite missing right lateral margin. **i** SUI 140015, fragment of exceptionally large tergite. T1–T5 = tergites 1–5, *SR* = scale row. Scale bars = 10 mm

**Fig. 16:**
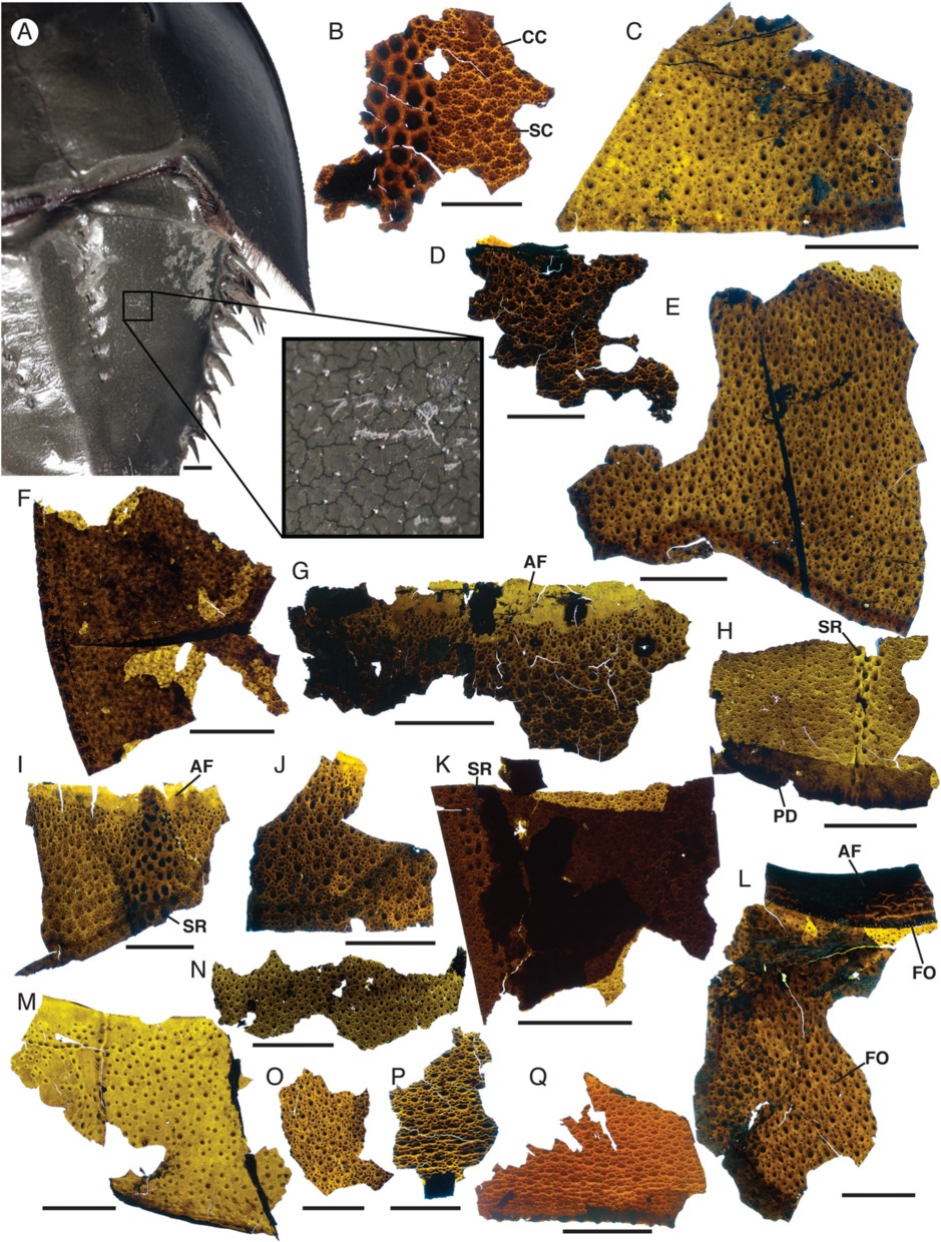
*Limulus polyphemus* and *Pentecopterus decorahensis*, cuticular features of tergites. **a** *Limulus polyphemus*, cracking of the cuticular surface. **b**–**q** *Pentecopterus decorahensis*, mesosomal tergites. **b** SUI 140020, portion of tergite showing row of enlarged scales and cracking of the cuticle. **c** SUI 140044, sparse scale ornamentation and cracking of the surface. **d** SUI 140039, weathered portion of cuticle showing advanced stages of cracking. **e** SUI 140046, regular scale ornament. **f** SUI 139954, lateral portions of articulated tergites. **g** SUI 140038, anterior portion of tergite showing the articulating facet devoid of ornamentation. **h** SUI 140027, tergite showing row of enlarged scales and posterior doublure. **i** SUI 140042, smooth anterior articulating facet and central row of enlarged guttalate scales. **j** SUI 140043, scale ornament. **k** SUI 140057, lateral portion of tergite showing ancillary row of scales along margin. **l** SUI 140045, anterior articulating facet of tergite. **m** SUI 140026, widely spaced follicles. **n** SUI 140028, uniform scale ornament. **o** SUI 140021, showing faint cracking of the cuticle. **p** SUI 140022, merging of scales towards posterior of tergite and fragment of posterior doublure. **q** SUI 140025, broadening and merging of rounded scales towards rear of tergite. *AF* = articulating facet, *CC* = cuticular cracking, *FO* = follicles, *PD* = posterior doublure, *SC* = scales, *SR* = scale row. Scale bars = 10 mm

**Fig. 17:**
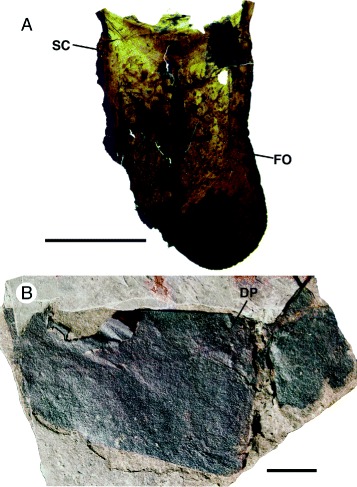
*Pentecopterus decorahensis*, genital appendage and operculum. **a** SUI 140003, genital appendage missing left lobe. **b** SUI 140008, incomplete genital operculum with left ala and deltoid plate. *DP* = deltoid plate, *FO* = follicles, *SC* = scales. Scale bars = 10 mm

**Fig. 18:**
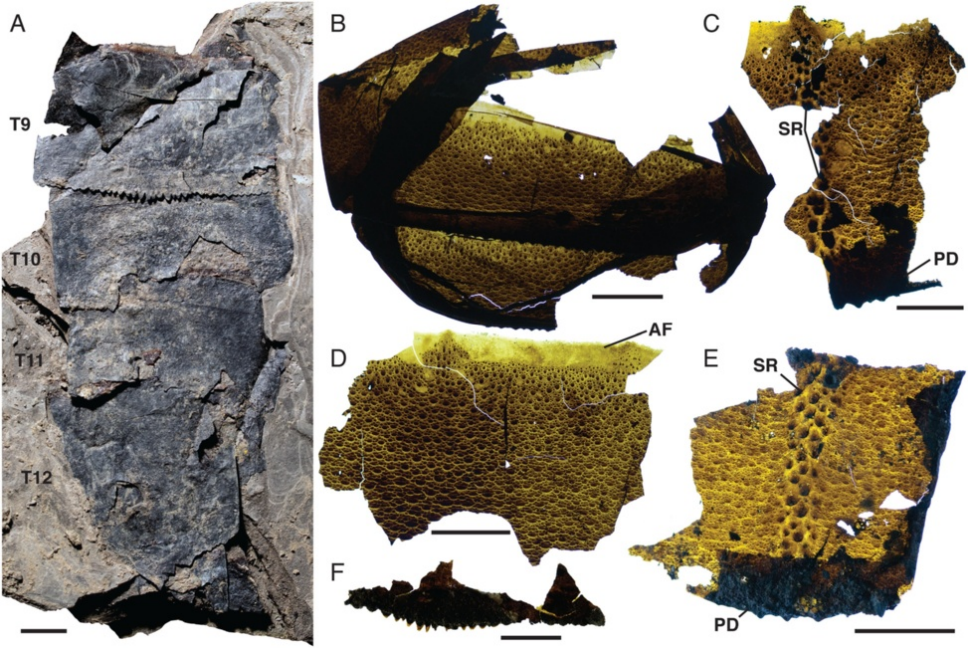
*Pentecopterus decorahensis*, metasomal tergites. **a** SUI 139955, tergites 9–12. **b** SUI 139976, tergites 8–9 showing dentate posterior margin. **c** SUI 139971, fragment displaying enlarged guttalate scale rows and posterior doublure with serrations. **d** SUI 140004, ventral cuticle with smooth articulating facet. **e** SUI 139974, cuticle showing central scale rows and posterior doublure. **f** SUI 140002, posterior margin of tergite with serrations. T9–T12 = tergites 9–12, *AF* = articulating facet, *PD* = posterior doublure, *PD* = posterior doublure, *SR* = scale row. Scale bars = 10 mm

**Fig. 19:**
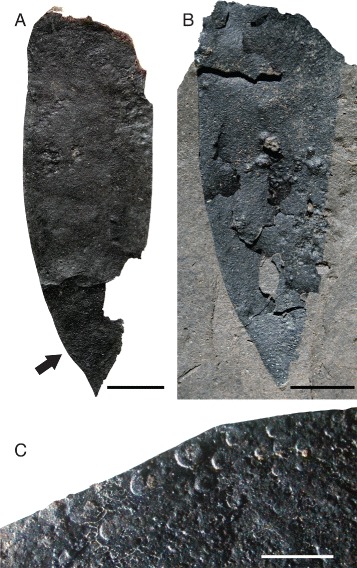
*Pentecopterus decorahensis* telson. **a** SUI 139956, almost complete, position of scales shown in Fig. 19c arrowed. **b** SUI 139957, showing partial exfoliation. **c** SUI 139956, detail of cuticular ornamentation. Scale bars for **a**–**b** = 10 mm, **c** = 2 mm

#### Etymology

The genus is named for the penteconter (Greek πεντηκόντορος), an early form of ancient Greek galley and one of the first true warships, which the taxon superficially resembles in outline and parallels in being an early predatory form. This is combined with -pterus (φτερός – wing), the epithet typically applied to eurypterid genera. The species name refers to Decorah in Winneshiek County, Iowa, where the material originates.

#### Material

Holotype: SUI 139941, prosomal ventral plate and proximal podomeres of prosomal appendage II. Paratypes: SUI 102857, SUI 139913, SUI 139917, SUI 139920, SUI 139924, SUI 139926, SUI 139931, SUI 139933, SUI 139935–139936, SUI 139945, SUI 139948, SUI 139953, SUI 139955–139956, SUI 139961, SUI 139965, SUI 139969, SUI 139979, SUI 139983–139984, SUI 139998–139999, SUI 140003, SUI 140008, SUI 140014. Additional Material: SUI 139912, SUI 139914–139916, SUI 139918–139919, SUI 139921–139923, SUI 139925, SUI 139927–139930, SUI 139932, SUI 139934, SUI 139937–139940, SUI 139942–139944, SUI 139946–139947, SUI 139949–139952, SUI 139954, SUI 139957–139960, SUI 139962–139964, SUI 139966–139968, SUI 139970–139978, SUI 139980–139982, SUI 139985–139997, SUI 140000–140002, SUI 140004–140007, SUI 140009–140013, SUI 140015–140061. Numerous fragmentary specimens in the University of Iowa Paleontology Repository.

#### Horizon and locality

Middle Ordovician (Darriwilian) Winneshiek Lagerstätte, Winneshiek Shale Formation, Winneshiek County, Iowa, USA.

#### Diagnosis

Megalograptidae retaining a single pair of spines on third podomere of prosomal appendages III; appendage V short with serrated distal margin of podomeres; prosomal ventral plates widening anteriorly; lateral margins of podomere VI-7 and VI-8 with small serrations; VI-7 with anterior rounded projection; pretelson lacking posterolateral expansion; telson xiphous, margin laterally ornamented with scales.

#### Description

The large number of fragmentary specimens of exuviae allows an almost complete description of the external morphology of the animal. The only structures that are not represented in the material are the prosomal shield and metastoma. A number of specimens represent juvenile instars (see discussion below).

The lack of specimens of the prosomal shield precludes any knowledge of the lateral eyes and ocelli, or of carapace cuticular ornamentation. The prosomal ventral plate is known from 12 specimens (Fig. 1) ranging from 60 mm to at least 144 mm in length and 62 mm to at least 122 mm in width (Table 1). The ventral plate is of *Erieopterus*-type, consisting of a single plate covering the anterior and lateral portion of the ventral carapace, as in modern horseshoe crabs. The ventral plate extends into a large rostrum anteriorly where no prosomal appendages could insert (Figs. 1c, f and 2); the appendages were attached to the soft ventral integument rather than the sclerotized ventral plates [14]. The appendages in SUI 139979 (Fig. 2) and the holotype SUI 139941 (Fig. 3) appear to have been displaced onto the rostrum during ecdysis as the animal pulled itself through the gap between the ventral plate and the carapace. The rostrum accounts for approximately half of the length of the ventral plates and has a shallow anterior indentation similar to that in *Waeringopterus* [54, 55] and *Eusarcana* [56, 57]. The rostral region of the ventral plate is drawn out posteriorly into a linguoid projection (Fig. 1d, e, f) which extends back between the prosomal appendage insertions (Fig. 3). A similar posterior process is present in *Erieopterus* [58] and would likely have projected between the chelicerae in life; this structure may be homologous to the ‘triangular area’ noted by Størmer [41] and Lamsdell [45]. Laterally, the ventral plate narrows evenly towards the posterior of the carapace (Fig. 1h) before terminating in an expanded lobe (Fig. 1a, f). The postero-lateral edge of this lobe folds dorsally over itself (SUI 139978, Fig. 1b) and this likely represents the posterior locking mechanism by which the ventral plate folds over onto the prosomal shield [14]. The lobed posterior outline of the ventral plate suggests that the carapace may have projected into genal facets, as the posterior margins of eurypterid ventral plates usually correspond closely to the posterior morphology of the prosomal shield [14]. Two specimens (Fig. 4) preserve fine cuticular details of the ventral plate and the ventral integument of the prosoma. The ventral plate cuticle is thick and preserved in a darker color with terrace lines and a row of scales along the interior margin, while the integument is flexible and covered in dense conical setae.

**Table 1:** *Pentecopterus decorahensis* prosomal ventral plate measurements

Specimen	Total length	Maximum width	Rostrum width	Main body length	Posterior process		Lateral projections	
					Length	Width	Length	Width
SUI 139914	42^a^	22^a^	–	–	–	–	42^a^	22
SUI 139915	64^a^	23^a^	–	–	–	–	64^a^	8
SUI 139916	144^a^	42^a^	–	61^a^	–	–	97	21
SUI 139917	60	62	29	28	5	4	32	7
SUI 139921	28^a^	24^a^	–	–	28	24	–	–
SUI 139936	66^a^	76^a^	35^a^	43	13	10	23^a^	19
SUI 139941	89^a^	111^a^	28^a^	57	19	14	16^a^	8^a^
SUI 139978	102^a^	40^a^	–	29^a^	–	–	72	13
SUI 139979	94^a^	122^a^	63^a^	52^a^	–	–	27^a^	21
SUI 139982	69^a^	33^a^	–	15^a^	–	–	54^a^	17
SUI 140009	54^a^	12^a^	–	–	–	–	54^a^	12^a^
SUI 140011	61^a^	21^a^	–	–	–	–	61^a^	21

All measurements in millimetres

^a^indicates an incomplete measurement

The sole near-complete specimen of the ventral plate (Fig. 1f, g) reveals that the general outline of the carapace was quadrate with a large anterior rostrum, i.e., an elongate trapezoidal outline. A clear marginal rim is present in some specimens (Fig. 2).

The most commonly preserved morphological features, excluding tergite fragments, are the prosomal appendages. All six appendages are represented, and a number of juvenile appendages are known. Appendages II–V are homonomous in juveniles (Fig. 5), each podomere bearing a single pair of ventral moveable spines and a pair of elongate fixed lateral spines projecting distally a length almost equal to that of the succeeding podomere. Each podomere is strongly denticulated distally towards its ventral edge. These juvenile appendages are densely ornamented with guttalate (droplet-shaped) scales (Fig. 6a) which are relatively larger and more closely spaced than in adult individuals (Fig. 6b, d, e). Appendage VI is similar to that in adults but appears to be relatively longer (see description of Appendage VI below).

The postoral appendages (II–VI) are differentiated from one another in adult specimens. Appendage I, the preoral chelicera (Fig. 7), comprises three segments: a non-spiniferous peduncle, a fixed finger, and a free finger; only six examples are known, all from adults. The peduncle of the chelicera (Fig. 7c) is approximately equal in length to the fixed ramus (Table 2), while the free ramus is about half this length (Fig. 7a, b). The free ramus terminates in a distal hook (Fig. 7d) which overlaps the termination of the fixed ramus (Fig. 7e). Neither ramus bears denticles (Fig. 7f). The second and third prosomal appendages are oriented anteriorly rather than ventrally, as shown by the rotation of the proximal podomeres, and bear enlarged armature, suggesting that their primary use was in prey capture. The morphology of appendage II is evidenced by seven specimens (Fig. 8), all but one of them adult (Table 3). Appendage II is relatively short with no more than seven podomeres but it is nonetheless robust and spinous. The coxa extends dorsally over the proximal podomeres of the endopod (Fig. 8c), increasing the area of the limb insertion into the body wall compared to that in most eurypterids, thereby effectively buttressing the appendage. Several coxae preserve a moveable endite (Fig. 8d). The paired ventral moveable spines on each podomere are conical and heavily sclerotized (Fig. 8a). The paired lateral fixed spines of podomeres four to six are enlarged compared to the width of the podomere (Fig. 8e, f). The paired lateral spines of the fourth podomere are angled ventrally (Fig. 3), extending in length almost to the distal termination of the appendage, and are serrated along the inner margin (Fig. 8b). Appendage III, in contrast, which is known from 17 specimens (Fig. 9), is a relatively simple raptorial limb (Fig. 9a, b), essentially similar but larger than the juvenile appendage (Table 4). The coxa is broad but has a narrow gnathobasic surface (Fig. 9a, c, i). The second podomere of the limb is modified to allow for greater rotation, with a wide, crescent-shaped distal aperture (Fig. 9c, j). The appendage armature is distinctly different from that of juvenile limbs; the paired ventral spines are largely reduced in size relative to the podomere width (Fig. 9d, n, o) although the lateral spines are enlarged and elongated (Fig. 9e, k, l), increasing in length through podomeres four to six. The penultimate podomere is long and circular in cross section, largely lacking in armature. The terminal podomere is a short, curved spine (Fig. 9f, g, h, m). Both these distalmost podomeres are usually obscured in lateral view by the massively elongate lateral spines of the sixth podomere (Fig. 9b).

**Table 2:** *Pentecopterus decorahensis* cheliceral measurements

Specimen	Appendage I
SUI 139934	(podomere 3): **Free finger**, 14/5.
SUI 139935	(podomeres 1–3): **Peduncle**, 15/8. **Fixed finger**, 17/7. **Free finger**, 9/4.
SUI 139939	(podomeres 1–3): **Peduncle**, 10^a^/4^a^. **Fixed finger**, 23^a^/11. **Free finger**, 8^a^/4.
SUI 139952	(podomeres 1–3): **Peduncle**, 28/11. **Fixed finger**, 28/12. **Free finger**, 14/5.
SUI 139972	(podomeres 2–3): **Fixed finger**, 13/5. **Free finger**, 4/2.
SUI 139983	(podomeres 2–3): **Fixed finger**, 28/12. **Free finger**, 15/5.

Length/width. All measurements in millimetres

^a^indicates an incomplete measurement

**Table 3:** *Pentecopterus decorahensis* prosomal appendage II measurements

Specimen	Appendage II
SUI 139919	(podomere 6): **6**, 14^a^/11.
SUI 139920	(podomeres 3–7): **3**, 15^a^/6^a^. **4**, 15/6^a^. **5**, 23/10. **6**, 21/10. **7**, 33/7.
SUI 139941	(podomeres 2–5): **2**, 15/23. **3**, 14/19. **4**, 20/18. **5**, 10^a^/7^a^.
SUI 139965	(podomeres 3–7): **3**, 2^a^/4. **4**, 3/4. **5**, 3/3. **6**, 2/2. **7**, 2/1.
SUI 139969	(podomere 1): **Coxa**, 9^a^/5.
SUI 139975	(podomeres 1–3): **Coxa**, 21/25. **2**, 9/14. **3**, 7^a^/12.
SUI 139979	(podomere 1): **Coxa**, 23^a^/12.

Length/width. All measurements in millimetres. Podomere identity indicated in bold

^a^indicates an incomplete measurement

**Table 4:** *Pentecopterus decorahensis* prosomal appendage III measurements

Specimen	Appendage III
SUI 102857	(podomeres 1–8): **Coxa**, 25^a^/52. **2**, 11/24. **3**, 16/19. **4**, 16/19. **5**, 19/17. **6**, 19/14. **7**, 12/7. **8**, 8/4.
SUI 139922	(podomere 6): **6**, 18^a^/17.
SUI 139925	(podomere 1): **Coxa**, 22^a^/21^a^.
SUI 139929	(podomeres 7–8): **7**, 20/8. **8**, 18/5.
SUI 139930	(podomeres 2–6): **2**, 15/27. **3**, 19/23. **4**, 16/20. **5**, 22/15. **6**, 18^a^/13.
SUI 139944	(podomeres 7–8): **7**, 17/7. **8**, 18/5.
SUI 139948	(podomeres 1–8): **Coxa**, 28^a^/30. **2**, 11/21. **3**, 21/20. **4**, 14/19. **5**, 20/13. **6**, 19/11. **7**, 18/8. **8**, 5^a^/4.
SUI 139951	(podomeres 1–5): **Coxa**, 27/23. **2**, 12/17. **3**, 14/13. **4**, 10/12. **5**, 15/8.
SUI 139952	(podomeres 1–5): **Coxa**, 35/21^a^. **2**, 17/26. **3**, 21/22. **4**, 15/18. **5**, 21/14.
SUI 139953	(podomeres 2–8): **2**, 15/19. **3**, 18/22. **4**, 16/19. **5**, 22/14. **6**, 22/23. **7**, 15/8. **8**, 22/7.
SUI 139963	(podomeres 1–8): **Coxa**, 7^a^/9^a^. **2**, 4/6. **3**, 5/6. **4**, 4/5. **5**, 4/5. **6**, 4/4. **7**, 4/3. **8**, 4/2.
SUI 139965	(podomeres 4–8): **4**, 2^a^/4. **5**, 4/4. **6**, 4/3. **7**, 5/3. **8**, 3/2.
SUI 139973	(podomere 6): **6**, 11^a^/13.
SUI 139979	(podomeres 1–4): **Coxa**, 34/23. **2**, 7/15. **3**, 8/15. **4**, 6^a^/16.
SUI 139990	(podomeres 7–8): **7**, 9^a^/9. **8**, 21/5.
SUI 140007	(podomeres 6–8): **6**, 15/14. **7**, 13/10. **8**, 12^a^/6.
SUI 140010	(podomere 1): **Coxa**, 34/31.
SUI 140013	(podomeres 6–8): **6**, 4^a^/5. **7**, 15/6. **8**, 8/4.

Length/width. All measurements in millimetres. Podomere identity indicated in bold

^a^indicates an incomplete measurement

The fourth to sixth prosomal appendages are shorter than the second and third and oriented ventrally. Appendage IV is known from 13 specimens (Fig. 10), the majority of which are isolated individual podomeres (Table 5). The most complete specimen (Fig. 10d) is attached to a portion of the carapace with a marginal rim, indicating that the marginal rim extended at least midway back along the carapace. As in appendages II and III, the coxa extends distally along the coxa-body junction (Fig. 10j), and ancillary rows of spinose hairs surround the gnathobases (Fig. 10i). Appendage IV is short (Fig. 10a), with fixed lateral spines extending parallel to the limb axis (Fig. 10c, d). The distal denticulations on each podomere are greatly developed and randomly oriented (Fig. 10e, g), forming an expanded (swollen) surface surrounding the base of the moveable ventral spines (Fig. 10b, f, h). The armature of Appendage V, which is known from 15 specimens (Figs. 11 and 12) (Table 6), is much less pronounced. The coxa bears a narrow gnathobasic surface with multiple rows of small teeth (Fig. 11g). The second podomere is curved ventrally (Fig. 11h, j, k). The margins of the distal podomeres are denticulate (Fig. 11c, e). The ventral spines are strongly reduced on all podomeres (Fig. 11d, f, l), as are the lateral spines on all but the penultimate podomere (Figs. 11b and 12a); the termination is trifurcate, made up of a terminal and two lateral spines (Fig. 11i). The overall morphology of Appendage V is slender compared to the more anterior appendages. This morphology of the fifth limb is similar to that of the equivalent appendage in *Megalograptus* [5] and *Eurypterus* [14], which is thought to have a ‘balancing’ function [4], but the podomeres are relatively shorter in adults (Fig. 11a) and the appendage flexes ventrally at the third podomere (Fig. 12b).

**Table 5:** *Pentecopterus decorahensis* prosomal appendage IV measurements

Specimen	Appendage IV
SUI 139926	(podomeres 5–8): **5**, 11/11. **6**, 10/10. **7**, 10/8. **8,** 21/5.
SUI 139927	(podomere 8): **8**, 21/5.
SUI 139928	(podomere 4): **4**, 12^a^/10.
SUI 139937	(podomere 3): **3**, 13/6.
SUI 139938	(podomere 3): **3**, 16/7.
SUI 139940	(podomeres 6–8): **6**, 7^a^/7^a^. **7**, 12/10. **8**, 13^a^/4.
SUI 139946	(podomeres 1–2): **Coxa**, 43/18^a^. **2**, 22^a^/17^a^.
SUI 139959	(podomere 1): **Coxa**, 14^a^/13.
SUI 139963	(podomeres 3–8): **3**, 5/5. **4**, 4/5. **5**, 5/4. **6**, 4/3. **7**, 2/2. **8**, 4/2.
SUI 139965	(podomeres 5–8): **5**, 5/4. **6**, 4/3. **7**, 5/2. **8**, 3/2.
SUI 139979	(podomeres 1–3): **Coxa**, 28/17. **2**, 13/15. **3**, 8^a^/12.
SUI 140000	(podomeres 2–3): **2**, 30^a^/21. **3**, 25^a^/20.
SUI 140012	(podomeres 6–8): **6**, 4^a^/7^a^. **7**, 9/9. **8**, 12^a^/6.

Length/width. All measurements in millimetres. Podomere identity indicated in bold

^a^indicates an incomplete measurement

**Table 6:** *Pentecopterus decorahensis* prosomal appendage V measurements

Specimen	Appendage V
SUI 139912	(podomeres 8–9): **8**, 16/9. **9**, 10/5.
SUI 139913	(podomeres 1, 6–9): **Coxa**, 19^a^/11^a^. **2**, −/−. **3**, −/−. **4**, −/−. **5**, −/−. **6**, 6^a^/10. **7**, 10/8. **8**, 10/7. **9**, 7/3.
SUI 139924	(podomere 1): **Coxa**, 30^a^/21.
SUI 139942	(podomeres 1–5): **Coxa**, 7^a^/10^a^. **2**, 6/4^a^. **3**, 8/8. **4**, 6/7. **5**, 6/6.
SUI 139968	(podomeres 6–9): **6**, 10/8. **7**, 9/6. **8**, 4/4. **9**, 4^a^/3.
SUI 139970	(podomeres 1–9): **Coxa**, 10^a^/12^a^. **2**, 4^a^/8. **3**, 2/7. **4**, 5/7. **5**, 5/7. **6**, 5/6. **7**, 6/5. **8**, 6/3. **9**, 4/2.
SUI 139979	(podomere 1): **Coxa**, 28/15.
SUI 139985	(podomeres 4–6): **4**, 20/17. **5**, 12/17. **6**, 14^a^/13.
SUI 139986	(podomeres 6–9): **6**, 5^a^/10. **7**, 7/8. **8**, 7/6. **9**, 11/4.
SUI 139989	(podomeres 3–4): **3**, 11^a^/12. **4**, 15/11.
SUI 139991	(podomeres 4–5): **4**, 11^a^/17. **5**, 9/10.
SUI 139996	(podomeres 4–5): **4**, 12/12. **5**, 10^a^/12.
SUI 139998	(podomeres 2–9): **2**, 6/18. **3**, 5/16. **4**, 12/15. **5**, 9/14. **6**, 10/12. **7**, 14/10. **8**, 7/6. **9**, 8/4.
SUI 140014	(podomeres 4–7): **4**, 20/18^a^. **5**, 13/21. **6**, 19/18. **7**, 16^a^/14.
SUI 140016	(podomeres 2–7): **2**, 3/6^a^. **3**, 3/10. **4**, 7/9. **5**, 5/7. **6**, 7/7. **7**, 7/6.

Length/width. All measurements in millimetres. Podomere identity indicated in bold

^a^indicates an incomplete measurement

Appendage VI, known from 24 specimens, expands distally into a paddle with an unusual morphology (Figs. 13 and 14) (Table 7). The expanded coxal gnathobasic surface is differentiated from the main body of the coxa by a narrow ‘neck’ (Fig. 13b). The gnathobase bears 16–18 teeth (Fig. 13a, c). The proximal podomeres of appendage VI are short and homonomous, distally serrated with a rounded ventral projection (Fig. 13d). The fourth podomere is longer than the third or fifth (Fig. 14c). The sixth podomere bears an enlarged ventral projection and is also expanded dorsally, resulting in a blade- or leaf-like shape (Fig. 13f, g). The distal margin of the sixth podomere bears elongate serrations that extend behind the ventral projection (Fig. 14i, k); podomere seven inserts into the region demarcated by these serrations (Fig. 14h), in line with the attachment point for the fifth podomere (Figs. 13e and 14e). The distal morphology of the sixth podomere is unusual compared to that in the equivalent appendage of other eurypterids; the anterior expansion allows for greater flexibility of the distal paddle (Fig. 14c). The seventh podomere is elongate and curved ventrally (Fig. 14d, j, m) with a serrated anterior margin and rounded distal projection (Fig. 14a). Podomere 7a is a small triangular element located ventrally on the distal margin of the seventh podomere and less than half its width (Fig. 14o), which overlaps podomere 8. The eighth podomere is oval, narrowing somewhat distally, and bears small lateral serrations (Fig. 14n) while the ninth podomere is short and narrow and inserts in a small recess in the posterior margin of podomere eight (Fig. 14b, f). In contrast to the scales that are present on the more proximal podomeres (Figs. 6f and 14l), podomeres 7–9 are ornamented with follicles, which increase in density towards the margins (Fig. 14e, g), and represent the insertions of sensory setae.

**Table 7:** *Pentecopterus decorahensis* prosomal appendage VI measurements

Specimen	Appendage VI
SUI 139918	(podomere 7): **7**, 42/22.
SUI 139923	(podomere 7): **7**, 52^a^/27^a^.
SUI 139933	(podomeres 7–9): **7**, 43/24. **7a**, 6/7. **8**, 13/12. **9**, 3/2.
SUI 139945	(podomere 1): **Coxa**, 40^a^/22^a^.
SUI 139949	(podomeres 1–3): **Coxa**, 23^a^/32^a^. **2**, 10/31. **3**, 11/25.
SUI 139958	(podomere 6): **6**, 17^a^/14^a^.
SUI 139960	(podomeres 5–7): **5**, 7^a^/13. **6**, 8/17. **7**, 28^a^/15.
SUI 139961	(podomere 6): **6**, 20/42.
SUI 139964	(podomeres 8–9): **8**, 17/8. **9**, 1/1.
SUI 139966	(podomeres 5–6): **5**, 7^a^/5^a^. **6**, 10^a^/16^a^.
SUI 139967	(podomere 6): **6**, 18/48.
SUI 139977	(podomere 7): **7**, 68/30.
SUI 139979	(podomeres 1–3): **Coxa**, 18^a^/26^a^. **2**, 6/19. **3**, 19/18.
SUI 139981	(podomeres 7–9): **7**, 74^a^/35. **7a**, −/−. **8**, 25^a^/20^a^. **9**, 10/4.
SUI 139984	(podomeres 1–7): **1**, 5^a^/5^a^. **2**, 10/13^a^. **3**, 13/14^a^. **4**, 21^a^/13^a^. **5**, 8/14. **6**, 11/30. **7**, 26^a^/21.
SUI 139987	(podomere 1): **Coxa**, 35^a^/29^a^.
SUI 139988	(podomere 7): **7**, 36^a^/17.
SUI 139992	(podomeres 8–9): **8**, 27^a^/21. **9**, 6/4.
SUI 139993	(podomere 6): **6**, 14/17^a^.
SUI 139994	(podomere 1): **Coxa**, 21^a^/20^a^.
SUI 139995	(podomeres 6–7): **6**, 6/9. **7**, 10^a^/10.
SUI 139997	(podomeres 5–6): **5**, 3^a^/18. **6**, 10/14^a^.
SUI 139999	(podomeres 7–9): **7**, 23^a^/18^a^. **7a**, −/−. **8**, 29^a^/18. **9**, 4/3.
SUI 140001	(podomeres 6–7a): **6**, 7/14. **7**, 33/13. **7a**, 3/3.

Length/width. All measurements in millimetres. Podomere identity indicated in bold

^a^indicates an incomplete measurement

The mesosoma is represented by a large number of specimens, the majority fragments of tergites (Figs. 15 and 16). Eleven specimens comprise relatively complete tergites or tergites in series (Table 8). The majority of specimens are incomplete laterally, but those that preserve the margins show no evidence of a lateral division (i.e., trilobation) (Fig. 15h) and they lack epimera (Fig. 15a, b). Some specimens are very large (Fig. 15g, i) but the width of these specimens does not greatly exceed the maximum carapace width as estimated from the ventral plate, suggesting that the eurypterid had a gracile outline. The length of the tergites increases evenly posteriorly (Fig. 15c, e, f) with the exception of the first tergite which is markedly reduced, as in other eurypterids [34]. The ornamentation of the mesosoma comprises a mixture of narrow and broad lunate scales interspersed with some enlarged guttalate scales (Fig. 16c, e, f, j, k, l, n, o, p, q). These guttalate scales, which are highly sclerotized, are arranged in three to four imperfect longitudinal rows along the center of each tergite (Fig. 16h, i). Follicles also occur across the cuticular surface (Figs. 15d and 16m). A smooth articulating facet extends across the anterior margin of each tergite (Fig. 16g, i). A pattern similar to desiccation cracks is evident on some specimens (Fig. 16b, d) and is likely due to taphonomic drying and shrinking of the cuticle; similar structures are found in carcasses of modern horseshoe crabs (Fig. 16a).

**Table 8:** *Pentecopterus decorahensis* mesosoma tergite measurements

Specimen	1	2	3	4	5	6
SUI 139931	–	–	–	50/123	60/88^a^	–
SUI 139932	–	–	–	–	–	70/85
SUI 139943	–	16/44^a^	15/40^a^	18/40^a^	–	–
SUI 139947	14/22^a^	22/41^a^	25/71^a^	26/43^a^	–	–
SUI 139950	–	–	–	70/106^a^	–	–
SUI 139954	–	19^a^/30^a^	16^a^/29^a^	–	–	–
SUI 139980	–	–	51^a^/100^a^	–	–	–
SUI 140005	–	–	–	69^a^/47^a^	–	–
SUI 140006	–	–	–	55^a^/68^a^	43^a^/65^a^	–
SUI 140015	–	–	–	–	104/140^a^	–
SUI 140017	–	–	–	–	33/54	44/54

Length/width. All measurements in millimetres. Isolated tergites were assigned to a segment based on size and differences in ornamentation

^a^indicates an incomplete measurement

Only a single specimen each of the genital operculum and genital appendage represent the ventral mesosomal structures. The operculum (Fig. 17b) is incomplete with a total preserved width of 72 mm, of which 55 mm is made up of the intact left lobe, and a length of 33 mm. The right lobe is too poorly preserved to show any details but the left lobe preserves a triangular deltoid plate, 15 mm long by 15 mm wide. The operculum preserves no evidence of an anterior opercular plate, nor of a suture or difference in ornamentation demarcating median and posterior plates. The genital appendage specimen consists of a single joint with a bilobate termination (Fig. 17a): it is unclear whether it represents a complete type B appendage or the distal joint of a type A appendage. The specimen is 24 mm long and 14 mm wide proximally, and the reconstructed distal width is 20 mm. A median suture line is present and a narrow doublure 2 mm wide runs along the lateral margins, expanding to 7 mm at the distal lobes. The genital appendage is ornamented proximally by small, outwardly-oriented scales, which give way distally to a dense covering of setal follicles.

The metasoma, which is comprised of the six posterior opisthosomal segments, is represented by seven specimens (Table 9) but only one of these preserves multiple articulated segments (Fig. 18a). The metasomal segments show no abrupt differentiation from those of the mesosoma, instead narrowing evenly from the seventh or eighth opisthosomal segment. The length of the metasomal segments decreases gradually to the pretelson, which is approximately 50 % longer than the preceding segment. The pretelson does not expand laterally and there is no evidence of an attachment point for cercal blades; this, combined with their absence in the material, indicates that such structures were lacking. The posterior margin of each metasomal segment is dentate (Fig. 18b, f), which distinguishes them from the mesosomal segments. The ornamentation of the metasomal and mesosomal segments is otherwise identical, the dorsal portion of both bearing a median row of highly sclerotized, enlarged scales (Fig. 18c, e). The ventral surface of the metasomal segments bears a similar ornamentation to the dorsal, with narrow angular scales in the anterior portion grading posteriorly into broad lunules with occasional chevrons. The ventral ornamentation is much more dense than the dorsal, however, and lacks the median row of enlarged scales (Fig. 18b, d). The metasomal segments show no evidence of epimera.

**Table 9:** *Pentecopterus decorahensis* metasoma tergite and telson measurements

Specimen	7	8	9	10	11	12	Telson
SUI 139955	–	–	32/58	28/55	28/51	40/46	–
SUI 139956	–	–	–	–	–	–	80/26
SUI 139957	–	–	–	–	–	–	70^a^/28
SUI 139962	–	20/82^a^	–	–	–	–	–
SUI 139971	–	49/34^a^	–	–	–	–	–
SUI 139974	–	–	–	–	–	29/29^a^	–
SUI 139976	–	19/61	16/52^a^	–	–	–	–
SUI 140002	12^a^/33^a^	–	–	–	–	–	–
SUI 140004	–	–	–	28^a^/41^a^	–	–	–

Length/width. All measurements in millimetres. Isolated tergites were assigned to a segment based on size and differences in ornamentation

^a^indicates an incomplete measurement

The telson is represented by two specimens (Fig. 19) and is xiphous, with a length/width ratio of between 2.5 and 3.0 (Table 9). The telson appears to lack a median ridge or keel (Fig. 19a, b), and is sparsely ornamented with broad lunules. The lateral margins are ornamented by heavily sclerotized scales similar to those forming median rows on the opisthosomal tergites (Fig. 19c).

#### Remarks

**Fig. 20:**
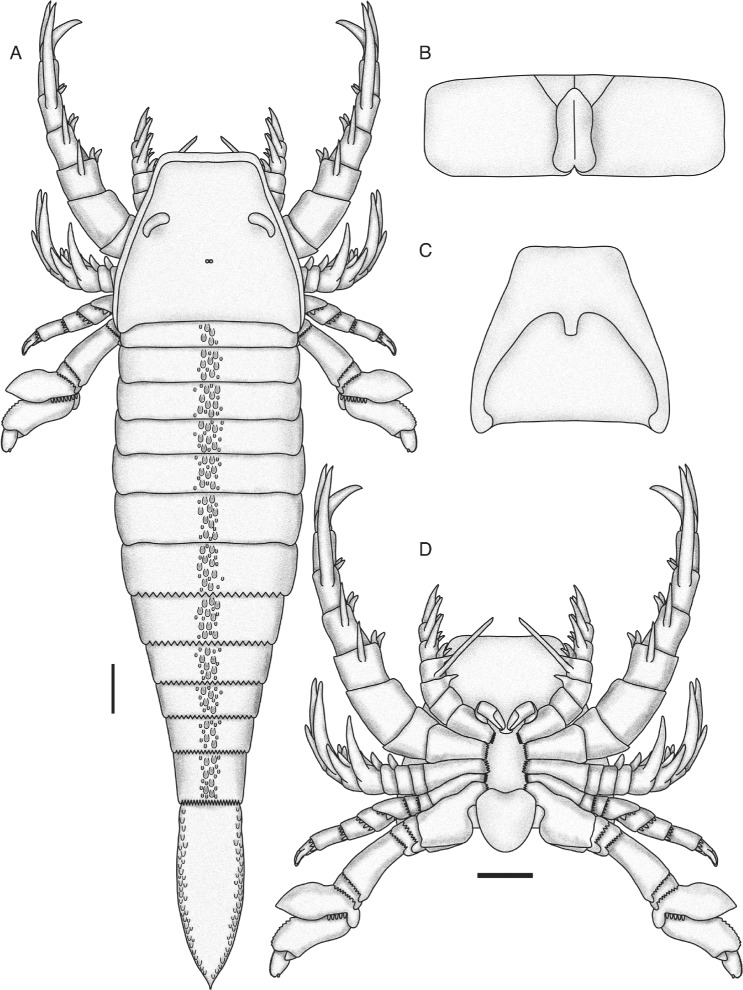
*Pentecopterus decorahensis*, reconstruction of adult. **a** Dorsal view. **b** Genital operculum. **c** Ventral view of carapace and prosomal ventral plate. **d** Ventral view of prosoma. The appendages are shown rotated in lateral view; in life, appendages IV–VI would be oriented so that the anterior edge (in IV) or the posterior edge (in V and VI) of the limb as in the reconstruction would face ventrally. The form of the median and lateral eyes and metastoma are hypothetical and based on ancestral state reconstructions using the phylogenetic matrix and topology. Scale bars = 10 cm (maximum size)

Despite the fragmentary nature of the material, the comprehensive representation of the morphology allows *Pentecopterus* to be reconstructed (Fig. 20). The taxon bears a number of similarities to *Megalograptus* [5], including the typical megalograptid guttalate ornamentation and a number of features of the prosomal appendages, notably the randomly-oriented armature on the distinctly swollen podomeres of appendage IV and the narrow gnathobase bearing multiple rows of small teeth on the coxa of appendage V, but it is nonetheless distinguished from *Megalograptus* by a number of characters (see Phylogenetic Affinities). *Pentecopterus*, like *Megalograptus*, bears rows of enlarged scales running down the center of the opisthosomal tergites but, unlike the scales in *Megalograptus*, those in *Pentecopterus* are not situated on pronounced ridges. *Pentecopterus* exhibits a number of morphological features unique within eurypterids, including the carapace shape and aberrant morphology of the sixth podomere of appendage VI. *Pentecopterus* is also unusual in the presence of lateral scales on the telson, a feature otherwise only observed in pterygotids [59]. However, the droplet-shaped guttalate scales in *Pentecopterus* differ from the angular chevron-type in pterygotids and the two conditions are likely convergent.

The size of *Pentecopterus*, from carapace anterior to telson posterior, can be inferred from individual fragmentary specimens based on the reconstruction. Most of the limb specimens indicate a total length of 75–100 cm, while the juvenile specimens indicate lengths of around 10–15 cm; some large tergites suggest lengths of up to 170 cm. This makes *Pentecopterus* the largest known megalograptid and by extension the largest known Ordovician eurypterid: at 85 cm, the average size of *Pentecopterus* outstrips the largest records of *Megalograptus ohioensis*, which ranges 49–78 cm in length [5]. Previous reports of megalograptids in excess of 200 cm in length are based on two fragmentary tergites of *Megalograptus shideleri* which Caster and Kjellesvig-Waering [5] considered to be derived from a giant individual based on the dimensions of cuticular scales. One of these tergites of *M. shideleri*, however, does not exceed 30 mm in length, suggesting a total body length of no more than 56 cm, far short of the sizes attained by *Pentecopterus*. Specimens of both *Pentecopterus* and *Megalograptus* show that scale size varies across the exoskeleton, with some scales being much larger than the average scale size.

Some features of *Pentecopterus* lend themselves to interpretations of the functional morphology and possible mode of life of the eurypterid. The second podomere of limbs II and III is modified to allow for greater rotation which, combined with the massive elongation of the ventrally-oriented spines, suggests that these limbs were angled forward in life and were involved primarily in prey capture rather than locomotion. The fourth to sixth prosomal appendages are shorter than the second and third, and oriented ventrally; their morphology suggests that they served a locomotory function resulting in a hexapodous gait. Ichnological evidence indicates that eurypterids adopted either a hexapodous [60–62] or octopodous [62, 63] mode of locomotion, although some trackways evidence a hexapodous gait with occasional transitions to octopodous locomotion [64, 65]. The interpretation of the gait of *Pentecopterus* as hexapodous is supported by trackways of the closely related taxon *Mixopterus* which exhibit a hexapodous gait [66]. The swimming capabilities of *Pentecopterus*, however, are difficult to determine. The sixth appendage is expanded into a paddle with an unusual morphology: the sixth podomere is drawn out and overlaps podomere seven laterally in much the same way as ‘podomere’ 7a overlaps podomere eight. This overlap likely increases the degree of movement possible at the podomere joint, as well as the surface area of the paddle, as has been hypothesized for ‘podomere’ 7a [14]. The enlarged denticulations on the distal margins of the podomeres would serve to lock them in place and reduce the degree of antero-posterior flexure of the paddle during forward and back strokes. This, combined with the increased paddle surface area, indicates that *Pentecopterus* was capable of swimming, although it has been suggested that the paddle of some eurypterids may have had a digging function [26, 45] and such a role for the paddle of *Pentecopterus* cannot be ruled out. *Pentecopterus* lacks the cercal blades that occur in *Megalograptus*, where they have been interpreted as functioning as a biological rudder, like the pterygotid telson [67]. Thus *Pentecopterus* may have been a less able swimmer than *Megalograptus*.

## Discussion

### Phylogenetic affinities

**Fig. 21:**
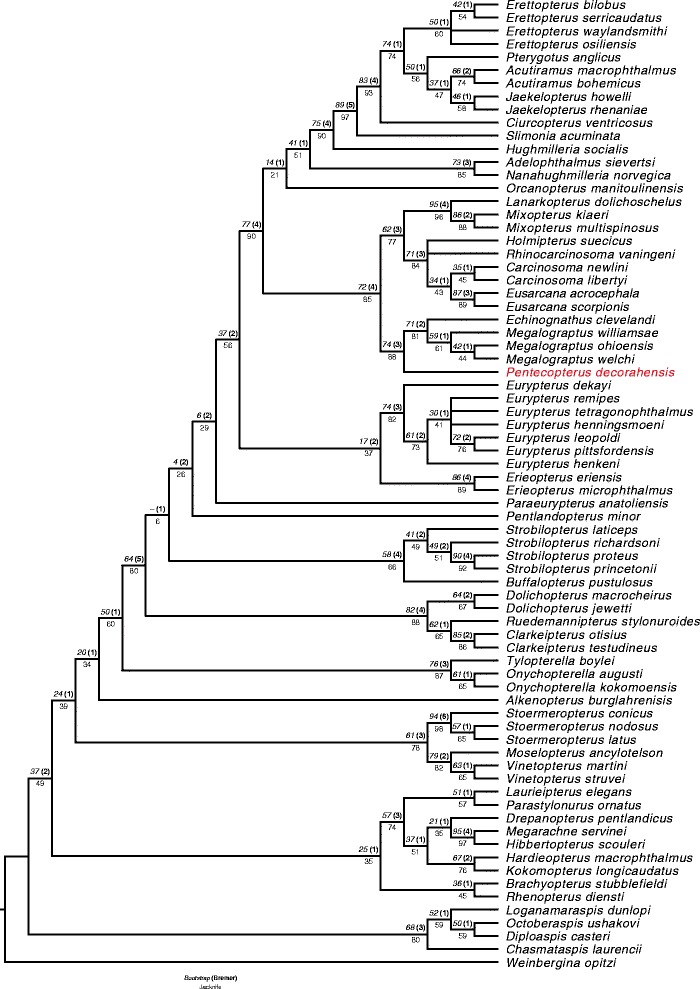
Result of the phylogenetic analysis*.* Single most parsimonious tree. Bootstrap branch support values are shown above the node in italics, with Bremer support values in bold within parentheses. Jackknife values are displayed beneath each node. The position of *Pentecopterus decorahensis* is highlighted in red. Arachnids are not included in the analysis

The phylogenetic analysis, as detailed in the methods section, yielded a single most parsimonious tree (Fig. 21) with a length of 475 steps, an ensemble Consistency Index of 0.429, ensemble Retention Index of 0.796, and Rescaled Consistency Index of 0.341. The topology is predominantly congruent with that retrieved from previous analyses of Stylonurina [68–70] and Eurypterina [9, 26, 45], with the exception of the position of *Alkenopterus*, which is retrieved here as a basal member of the Eurypterina as suggested by Poschmann [46]. *Pentecopterus decorahensis* is resolved as the basalmost member of the Megalograptidae, united with *Echinognathus* and *Megalograptus* by the shared possession of two or more pairs of spines per podomere on prosomal appendage IV, a reduction of all spines of appendage V except the pair on the penultimate podomere, an ornamentation of angular scales across the posterior margin of the dorsal tergites, longitudinal rows of large scales on the tergites, and an ornamentation consisting predominantly of guttalate scales. *Pentecopterus* is separated from the other genera in the Megalograptidae (*Echinognathus* and *Megalograptus*) by the presence of only one pair of spines on the third podomere of appendage III, a single terminal spine on each prosomal appendage, and the absence of dense cuticular ornamentation.

Megalograptidae resolves as a basal clade of Carcinosomatoidea, which also includes the families Mixopteridae and Carcinosomatidae, the superfamily being united by an anterior carapace projection, enlargement of the forward appendages, elongation of the spines on appendage III, an anterior rather than ventral orientation of prosomal appendages II and III, and the presence of thickened and highly sclerotized spines on appendages II–IV. This grouping was first proposed by Størmer [71] and was considered to be a relatively derived clade of eurypterids by Novojilov [72]; however, in their revision of the group, Caster and Kjellesvig-Waering [5] considered *Megalograptus* to be an extremely primitive eurypterid while regarding it as related to the carcinosomatoids. The hypothesis that *Megalograptus* exhibits ‘primitive’ (i.e., plesiomorphic) characteristics, combined with its early stratigraphic occurrence, led Tetlie [73] to consider megalograptids as a clade distinct from carcinosomatoids occupying a basal position within the eurypterids, while carcinosomatoids remained as a relatively derived group having attained a number of shared characteristics with megalograptids through convergence. As such, *Megalograptus* has been considered to represent the plesiomorphic condition within eurypterids for a number of characters, including the occurrence of the opisthosomal preabdominal/postabdominal constriction at the sixth and seventh segment as opposed to the seventh and eighth [5], the possession of an extra podomere on prosomal appendages II–V [72], and the morphology of the prosomal ventral plates [37]. Nevertheless no phylogenetic analysis has retrieved megalograptids in such a basal position: *Megalograptus* has either been excluded from consideration due to its mix of plesiomorphic, derived and autapomorphic characteristics [74] or has resolved as part of a derived clade of carcinosomatoids [9, 26, 45]. The analysis presented herein provides strong support for Megalograptidae as part of a carcinosomatoid clade. Furthermore, the presence of *Pentecopterus* at the base of the megalograptids reveals that a number of supposedly plesiomorphic characters in *Megalograptus* are either reversals or derived conditions. A single character in particular, the lack of a modified distal margin of the sixth podomere of the swimming leg, was used to infer a basal position for *Megalograptus* [73]. The morphology of the sixth appendage of *Pentecopterus* is somewhat unusual and resembles that of *Strobilopterus* [75] but the distal margin of its sixth podomere is clearly modified, indicating that the unmodified condition in *Megalograptus* is a reversal. Similarly, the unique arrangement of the prosomal ventral plates in *Megalograptus* appears to be derived from the anterior carapace projection folding ventrally over the *Erieopterus*-type ventral plates of *Pentecopterus. Pentecopterus* also demonstrates that the fifth ‘balancing’ limb of *Megalograptus* is independently derived from that of *Eurypterus*, as the adult morphology of appendage V in *Pentecopterus* exhibits the reduction of spines but lacks the elongated, tubular podomeres of *Megalograptus* and *Eurypterus*.

### Implications for early eurypterid evolution

**Fig. 22:**
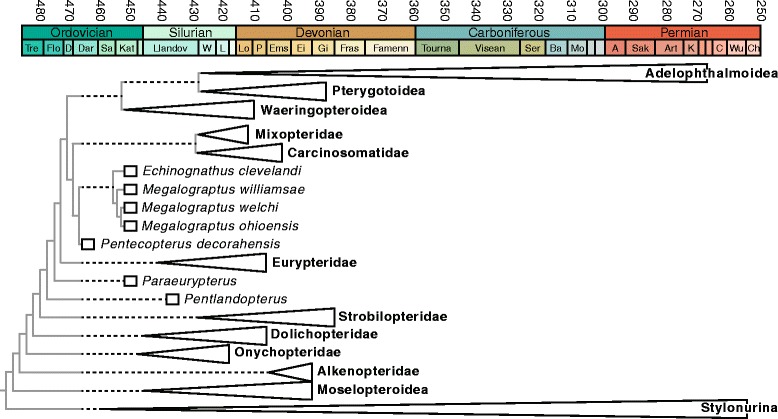
Evolutionary tree of the major clades of Eurypterina. Stratigraphic ranges of clades are shown by triangles, those of individual genera and species by boxes. Dashed black lines represent inferred ghost ranges. Solid gray lines show the tree topology

*Pentecopterus decorahensis* is the oldest described eurypterid, predating *Brachyopterus stubblefieldi* from the Sandbian of Avalonia [7] by some 9 million years. Eurypterids from the Tremadocian Fezouata formations of Morocco await investigation [76]. Several eurypterid clades have already been identified with long ghost ranges extending into the Upper Ordovician [9] but the placement of megalograptids within Carcinosomatoidea increases the number of clades with ranges that must have extended into the Middle Ordovician (Fig. 22). The inferred appearance of a large number of morphologically diverse eurypterid clades during the Darriwilian indicates that the eurypterids radiated explosively during the early stages of the Ordovician or that they originated during the Cambrian and underwent a period of cryptic evolution prior to the Ordovician radiation. The former scenario is supported by the observation that clades commonly reach their maximum disparity early in their evolution [77] and rates of morphological change are greater during this interval [78]. However, chasmataspidids, which form the sister group to the clade comprising eurypterids and arachnids [34], may range back to the Upper Cambrian [79], suggesting that eurypterids may also originate prior to the Ordovician.

It has been suggested that eurypterids originated in Gondwana, as the Ordovician eurypterids from Gondwana are basal forms with poor dispersal abilities, whereas those from Laurentia are more derived forms capable of active swimming [9]. As a swimming form, like other megalograptids [67], *Pentecopterus* is consistent with this pattern. All early Laurentian eurypterids are relatively large predators, either megalograptids [5] or waeringopterids [6]. The taxonomically restricted nature of the Laurentian fauna may be due to a limited influx of early colonists from Gondwana, whereas other eurypterid groups arrived in the early Silurian.

### Eurypterid cuticular structures

**Fig. 23:**
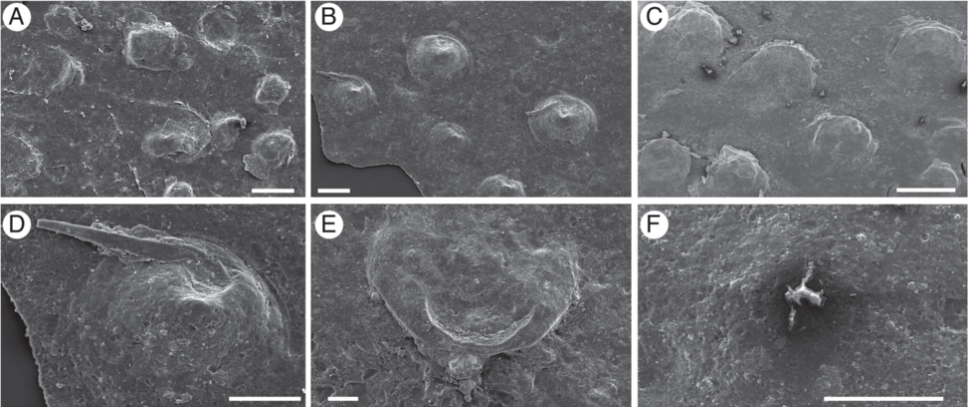
*Pentecopterus decorahensis,* SEM images of cuticular structures. **a** SUI 139963, scales on juvenile appendage. **b** SUI 139951, scales and setae on coxa of appendage III. **c** SUI 140020, scales on mesosomal tergite. **d** SUI 139951, scale and seta on appendage III coxa. **e** SUI 140031, scale on mesosomal tergite showing base of seta projecting from beneath the posterior margin. **f** SUI 140054, follicle on mesosoma with base of seta. Scale bars for **a**, **b**, **e** = 100 μm, **c** = 1 mm, **d**, **f** = 50 μm

The exceptional preservation of the cuticle of *Pentecopterus* allows for a preliminary study of cuticular structures. As in the majority of more derived eurypterids [9, 45], scales are the primary ornamentation, occurring on the prosomal appendages (Fig. 6), mesosomal and metasomal segments (Figs. 16 and 18), and the telson (Fig. 19). The scales, which formed through thickening and folding of the cuticular surface (Fig. 23a, c), are predominantly uniform in size. Notable exceptions are the large scales which occur in a single dorsal row on each prosomal appendage, and the darker thicker ones, presumably highly sclerotized, which form central rows on the opisthosomal tergites. Many of the smaller scales bear a follicle at their apex. The morphology of these scales falls into two distinct categories: those on the coxae of the prosomal appendages are conical, do not extend far over the underlying limb podomere, and bear a seta located in a notch beneath the apex of the cone (Figs. 6c and 23b, d); those on the opisthosoma bear setal bases that project from beneath the scale where it overlaps the tergite below (Fig. 23e).

Numerous isolated follicles with broken setal bases are preserved on the opisthosomal cuticle surface (Fig. 23f); these follicles are also prevalent on the prosomal appendages, where they appear to be concentrated in the ventral region of the podomeres (Fig. 12). Setal density is particularly high on the paddle of the sixth appendage where it increases towards the paddle margin (Fig. 14e) forming a fringe of setae similar to that in brachyuran swimming crabs [80]. In brachyurans these setae are stiff and function to expand the surface area of the paddle during swimming [80]. The follicles in eurypterids are smaller than in brachyurans and the setae may have had a sensory function.

**Fig. 24:**
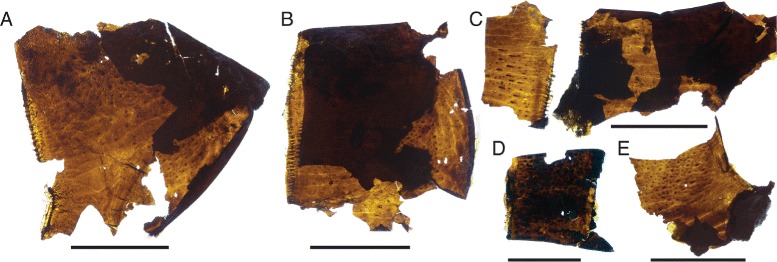
*Pentecopterus decorahensis,* prosomal appendage cuticle. **a** SUI 140049, podomere III of appendage IV showing fringe of setae around margin. **b** SUI 140049, podomere of appendage IV showing distal fringe of setae. **c** SUI 140051, podomere of appendage V, part and counterpart. **d** SUI 140019, podomere of juvenile appendage showing distal denticulations and moveable spine. **e** SUI 140050, portion of podomere cuticle. Scale bars = 1 mm

Fringes of short, broad setae also surround the distal margin of the podomeres of the prosomal appendages (Fig. 24a, b, c), where they have been recorded previously in *Eurypterus* [14]. These setae occur in the same position as the marginal denticulations of the distal limb podomeres (Fig. 24d) and these two structures may be homologous. Ancillary spines, similar in form but much larger and more robust than the setae present on the rest of the limb, are evident on the coxa of appendage II (Fig. 8d) and appendage IV (Fig. 10i); similar spines occur on the coxae of xiphosurids [81], where they aid in food mastication.

Setae are also preserved on the integument surrounding the prosomal ventral plate (Fig. 4b, c). Here they take the form of very fine hairs covering the cuticle on the ventral surface of the prosoma where the appendages and ventral plate insert. Similar setation is present in modern horseshoe crabs [81]. The ventral plate itself bears a row of scales along its inside margin (Fig. 4a) and terrace lines (Fig. 4b) across the remainder of the plate. Terrace lines have been reported in a number of other eurypterids [14, 45, 82] and are prevalent in trilobites [83] and decapod crustaceans [84, 85]. Setae often insert along the terrace lines in trilobites [86], and such setae were likely sensory in both trilobites and eurypterids [45, 86].

### Appendage differentiation and ontogeny

The smallest eurypterid specimens (SUI 139963 and SUI 139965, Fig. 5) exhibit an appendage morphology different from that of the larger individuals. They bear a series of homonomous appendages but the armature, which consists of a pair of moveable ventral spines, a pair of elongate fixed lateral spines, and a denticulate distal margin on each podomere, shows marked similarities to that in the large *Pentecopterus* specimens. All the appendages share a distinctive ornament of guttalate scales, which are relatively larger and more densely spaced in the smaller individuals. The number of scales in the smallest specimens is similar to that in the larger ones, although the distance between them increases. The occurrence of a relatively small ventral plate (Fig. 1f, g) confirms that small individuals of *Pentecopterus* occur at the locality, even though no small specimens showing the morphology of the larger appendages are present. The similarities in armature and ornamentation, combined with the absence of any other morphologically distinct eurypterid material in the fauna, suggests that all the available Winneshiek eurypterid material represents the same species and that SUI 139963 and SUI 139965 are juvenile individuals of *Pentecopterus decorahensis*.

Juveniles have been reported for only a handful of eurypterid species [4, 25, 26, 70, 87]. In-depth analysis of allometric trends between instars has only been attempted for *Eurypterus remipes* [23–25], *Hardieopterus* (?) *myops* [88], *Adelophthalmus luceroensis* [87], and *Strobilopterus proteus* [26]. Such a detailed study is not possible for *Pentecopterus*, as there are only two readily identifiable juveniles. *Pentecopterus* does, however, reveal a degree of ontogenetic change in appendage armature previously undocumented in eurypterids. Change in the morphology of the sixth appendage paddle has been described in *Strobilopterus princetonii* [75] and an allometric decrease in relative appendage length was noted in *Drepanopterus pentlandicus* [70] and *Strobilopterus proteus* [26], but the configuration of the appendage armature does not vary between instars in any of these taxa. *Pentecopterus*, in contrast, undergoes change in appendage armature as well as an allometric shift in appendage proportions.

**Fig. 25:**
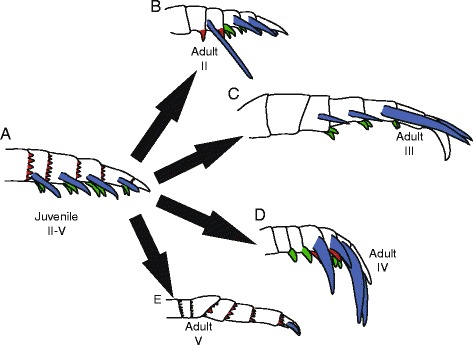
Ontogenetic changes in prosomal appendages of *Pentecopterus decorahensis* from juvenile to adult (coxa not shown). **a** Juvenile appendages II–V. **b** Adult appendage II. **c** Adult appendage III. **d** Adult appendage IV. **e** Adult appendage V. Homologous structures are color coded: red = distal denticulations, green = moveable ventral spines, blue = fixed lateral spines

Appendages II–V appear homonomous in juvenile *Pentecopterus*: each podomere shows a strong denticulation of the distal margin, and bears a fixed lateral spine pair extending the length of the succeeding podomere, as well as a moveable ventral spine pair equal in length to the width of the podomere (Fig. 25a). In adult specimens (Fig. 25b, c, d, e), in contrast, the distal denticulation is reduced in appendages II and III but unmodified in appendage V, while the denticulations in appendage IV migrate to the ventral margin of the podomere where they are enlarged into multiple fixed spines located on a swollen surface bearing the moveable ventral spines. The ventral spines are unmodified in appendage IV, but are almost vestigial on appendage III and completely lost on appendage V. In contrast, the ventral spines on appendage II are robust and highly sclerotized, retaining their length relative to podomere width but increasing their width at the base of the spine. The lateral spines of appendage II also undergo some differentiation: they appear unmodified on the distal podomeres, whereas those on the third podomere are oriented ventrally and dramatically extended to a length almost equal to that of all the succeeding podomeres combined (Fig. 8e). The lateral spines of appendages III and IV are not much modified. Those on appendage III become gradually longer from podomeres four to six but are absent on the seventh, penultimate podomere. Appendage V shows the opposite pattern; lateral spines are absent on all podomeres except the penultimate podomere 8. Appendage VI is very similar in juveniles and adults but the juvenile paddles are more gracile (Fig. 14h, o), with the adult paddle exhibiting greater overlap between the sixth and seventh podomeres (Fig. 14c) and a relatively shorter seventh podomere (Fig. 14a). The relative decrease in paddle length in adults may indicate that juveniles were more able swimmers, although the greater overlap between the sixth and seventh podomeres in adults suggests that the surface area of the appendage could be modified to a greater extent during swimming.

Appendage morphology is used as a key diagnostic feature for assigning eurypterid species to higher clades [44] and differences in armature would normally be considered indicative of a separate species. Our demonstration that at least some eurypterid species undergo changes in armature morphology during ontogeny reinforces the importance of including ontogenetic data when defining and describing taxa [89]. Division of form and function in the prosomal appendages has been considered a significant character in defining eurypterid higher taxa, and is thought to display strong evolutionary signal [41, 44]. A lack of appendage differentiation could result in juvenile eurypterids, like that of *Pentecopterus*, being placed in more basal eurypterid clades if their juvenile nature was not recognized.

**Fig. 26:**
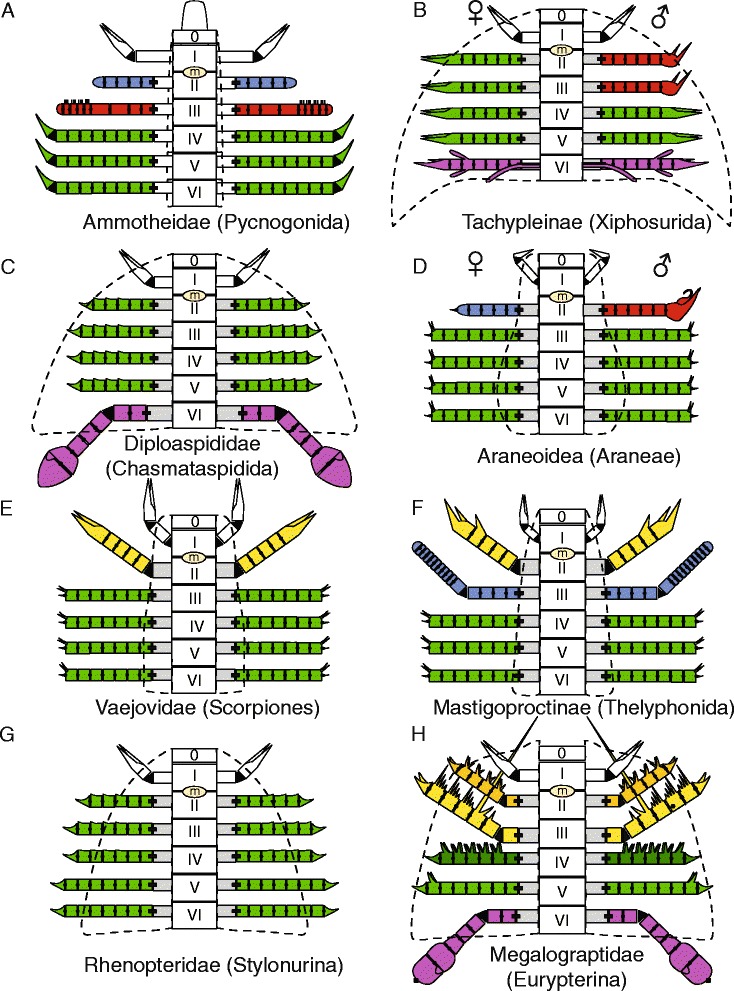
Appendage specialization and differentiation among chelicerate clades. **a** Pycnogonids (sea spiders). **b** Horseshoe crabs. **c** Chasmataspidids. **d** Spiders. **e** Scorpions. **f** Whip scorpions (vinegaroons). **g** Stylonurine eurypterids. **h** Eurypterine eurypterids. Somites are labeled 0–VI; ‘m’ indicates the position of the mouth. Appendages are color coded by morphology and function: green = locomotory, red = modified for reproduction, blue = tactile/sensory function, yellow = raptorial, purple = modified for a swimming/digging. For Megalograptidae, darker shades of yellow and green indicate limbs with a raptorial and locomotory function respectively but with a different morphology to other limbs in the taxon with a similar function. Where sexual dimorphism is present the condition in the female is shown on the left and the male on the right

Functional differentiation of the prosomal appendages is known in all major chelicerate groups, with a tendency to modification of the second appendage (the pedipalps) for feeding or reproduction (Fig. 26) [90]. The third appendage is modified in some pycnogonids and xiphosurids for use in reproduction, as either an ancillary pair of claspers or ovigers (Fig. 26a, b), and this appendage is differentiated into a tactile sensory limb (Fig. 26f) in some arachnids, including thelyphonids, while some opilionids have a differentiated fourth appendage. The sixth prosomal appendage is modified in both xiphosurids and chasmataspidids for use in locomotion or burrowing (Fig. 26b, c). Some eurypterids show an extraordinary degree of differentiation, with megalograptids exhibiting differentiation of every appendage pair (Fig. 26h), but the appendages of the basal-most eurypterids are either undifferentiated or exhibit differentiation of the posterior appendage pair only (Fig. 26g). Eurypterids differ from many other chelicerates in undergoing a postembryonic differentiation of appendages other than those used in reproduction; pycnogonids undergo no postembryonic differentiation of the appendages except for the ovigers [91], and the limbs in xiphosurids remain unchanged except for the differentiation of the claspers in males [92]. Arachnids, in contrast, frequently exhibit post-larval differentiation of the pedipalps for both prey capture [93, 94] and reproduction [95, 96]. The strong degree of appendage differentiation in eurypterids reflects their close phylogenetic relationship to arachnids [34].

## Conclusions

The newly described eurypterid *Pentecopterus decorahensis* from the Winneshiek Lagerstätte is the earliest described representative of the group, pushing our knowledge of Eurypterida back some 9 million years to the Darriwilian in the Middle Ordovician. *Pentecopterus* shows clear affinities with megalograptids, a highly distinct group of large predatory eurypterids known solely from the Ordovician of North America. Inclusion of the taxon in an expanded phylogenetic analysis of Eurypterida resolves *Pentecopterus* as basal within the megalograptid clade, which is itself part of the relatively derived carcinosomatoids. *Pentecopterus* reveals that a number of characteristics thought to link megalograptids with basal Eurypterina, such as the absence of a modified distal margin to the sixth podomere of the paddle, represent reversals in *Megalograptus*. Meanwhile, characters that were regarded as indicative of affinities between *Megalograptus* and *Eurypterus*, such as modification of the fifth prosomal appendage into a ‘balancing’ limb, are due to convergence. The occurrence of derived eurypterid clades in the Middle Ordovician indicates that Eurypterida either have a longer evolutionary history than previously recognized, extending back into the Cambrian, or underwent an explosive radiation following an Ordovician origin. This would also push back the origin of arachnids, which likely have a sister-group relationship with eurypterids [34, 35] from the early Silurian to at least the Middle Ordovician.

As well as informing on broader evolutionary trends, numerous specimens of *Pentecopterus* reveal the patterning and structure of the cuticular ornament, including scales and setal insertions, allowing for direct comparison with exceptionally preserved material of Silurian age *Eurypterus* [14]. This allows for potential exploration of general properties of eurypterid cuticle and ornamentation, as well as revealing the position of sensory setae and the form of podomere articulations. The identification of juvenile specimens of *Pentecopterus* provides evidence for an unusual degree of postembryonic appendage differentiation in eurypterids. Initial studies utilizing eurypterid phylogeny [1] and exquisitely preserved morphological features [97] such as those reported here are already beginning to place eurypterids within an ecological framework, and this diverse arthropod group represents a promising source for macroevolutionary and macroecological studies.

### Availability of supporting data

The data set supporting the results of this article is available in the MorphoBank repository, http://morphobank.org/permalink/?P2116, and is also included within the article and its additional file.

## Additional file

Additional file 1:
**Data matrix for the phylogenetic analysis.** (NEX 35 kb)
